# Characterization of disease-propagating stem cells responsible for myeloproliferative neoplasm–blast phase

**DOI:** 10.1172/jci.insight.156534

**Published:** 2022-04-22

**Authors:** Xiaoli Wang, Raajit K. Rampal, Cing Siang Hu, Joseph Tripodi, Noushin Farnoud, Bruce Petersen, Michael R. Rossi, Minal Patel, Erin McGovern, Vesna Najfeld, Camelia Iancu-Rubin, Min Lu, Andrew Davis, Marina Kremyanskaya, Rona Singer Weinberg, John Mascarenhas, Ronald Hoffman

**Affiliations:** 1 Division of Hematology/Medical Oncology/Pathology, Tisch Cancer Institute, Icahn School of Medicine at Mount Sinai (ISMMS), New York, New York, USA.; 2 Center for Hematologic Malignancies, Memorial Sloan Kettering Cancer Center, New York, New York, USA.; 3 Genetics and Genomic Sciences, ISMMS, New York, New York.; 4 Sema4, Stamford, Connecticut, USA.; 5 New York Blood Center, New York, New York, USA.

**Keywords:** Oncology, Stem cells, Hematopoietic stem cells, Leukemias

## Abstract

Chronic myeloproliferative neoplasms (MPN) frequently evolve to a blast phase (BP) that is almost uniformly resistant to induction chemotherapy or hypomethylating agents. We explored the functional properties, genomic architecture, and cell of origin of MPN-BP initiating cells (IC) using a serial NSG mouse xenograft transplantation model. Transplantation of peripheral blood mononuclear cells (MNC) from 7 of 18 patients resulted in a high degree of leukemic cell chimerism and recreated clinical characteristics of human MPN-BP. The function of MPN-BP ICs was not dependent on the presence of *JAK2V617F*, a driver mutation associated with the initial underlying MPN. By contrast, multiple MPN-BP IC subclones coexisted within MPN-BP MNCs characterized by different myeloid malignancy gene mutations and cytogenetic abnormalities. MPN-BP ICs in 4 patients exhibited extensive proliferative and self-renewal capacity, as demonstrated by their ability to recapitulate human MPN-BP in serial recipients. These MPN-BP IC subclones underwent extensive continuous clonal competition within individual xenografts and across multiple generations, and their subclonal dynamics were consistent with functional evolution of MPN-BP IC. Finally, we show that MPN-BP ICs originate from not only phenotypically identified hematopoietic stem cells, but also lymphoid-myeloid progenitor cells, which were each characterized by differences in MPN-BP initiating activity and self-renewal capacity.

## Introduction

The Philadelphia chromosome–negative myeloproliferative neoplasms (MPN), including polycythemia vera (PV), essential thrombocythemia (ET), and primary myelofibrosis (PMF), are clonal hematopoietic stem cell (HSC) neoplasms characterized by the proliferation of terminally differentiated myeloid cells (1). Approximately 1%, 4%, and 20% of ET, PV, and PMF patients, respectively, progress to a leukemic or blast phase (BP) termed MPN-BP over a 10-year period following diagnosis (2). Patients with MPN-BP have 20% or more leukemic blasts in the peripheral blood (PB) or BM (3). MPN patients with leukemic blasts between 10% and 19% in the PB or BM are classified as being in accelerated phase (AP) disease and have also been shown to have poor outcomes relative to chronic phase (CP) patients (3, 4). Patients with MPN-BP have a particularly dismal prognosis with a median overall survival (OS) ranging from 2.6 to 8.0 months with currently available therapies (3, 5–6). Moreover, MPN-BP is almost uniformly resistant to induction chemotherapy or strategies including hypomethylating agents (4–6). Therefore, there is an unmet need for novel therapeutic strategies capable of eliminating MPN-BP stem cells (SC) and/or delaying/preventing progression of chronic MPNs to MPN-BP. The development of such strategies relies on the comprehensive understanding of molecular and cellular biology of MPN-BP SCs, which, to date, has not been systemically studied.

*JAK2V617*, as well as mutations in calreticulin (*CALR*) and the thrombopoietin (TPO) receptor *MPL*, are the most common driver mutations associated with the chronic MPNs (3, 7). Mutations in *TP53*, *IDH1/2*, *RUNX1*, *ASXL1*, *EZH2*, and *SRSF2* are predictive of the evolution of a chronic MPN to MPN-BP (8–13). In addition, alterations in the cellular epigenetic profile, as well as specific cytogenetic events, are associated with the development of MPN-BP (14). Mutational analyses of paired samples before and after the development of BP have led to the identification of 2 possible routes by which a CP MPN might progress to MPN-BP: (a) the emergence of MPN-BP clones that are distinct from the original MPN clone or (b) clonal progression of the chronic MPN clone to MPN-BP due to the acquisition of additional genetic mutations (12, 15–16). Leukemic transformation (LT) of the chronic MPNs has previously been shown to be driven by the acquisition of distinct time-dependent molecular events (17). However, these previously reported observations have been largely made by sequencing bulk primary MPN and MPN-BP cells. At the time of LT, blood and BM samples contained cells representing the leukemic process, as well as others derived from the prior chronic MPN. As a result, these observations may not accurately reflect the molecular characteristics of MPN-BP. The genomic architecture of MPN-BP SCs has been especially poorly characterized. Moreover, little is known about how various SC subclones evolve or their impact on the unusually aggressive clinical nature of MPN-BP. We hypothesize that MPN-BP may be the consequence of multiple SC clones/subclones with different genetic mutations and cytogenetic abnormalities that may differ in their contribution to the evolution to MPN-BP.

Leukemia stem cell (LSC) clones have been reported to originate from distinct cells along the hematopoietic developmental hierarchy that display considerable differences in proliferative and differentiation potential, self-renewal capacity, and responses to therapy (18–22). In MPN-BP, studies of a *JAK2V617F*/*TP53*-mutant MPN-BP mouse model demonstrated that the leukemic clone appeared to arise from an aberrant megakaryocyte-erythrocyte progenitor (MEP) cell population (12). However, the cell of origin of MPN-BP SCs in primary patients with MPN-BP has not been documented.

In this study, we, therefore, have used an MPN-BP initiating cell (IC) serial xenograft system to phenotypically, functionally, and genotypically examine the cells of origin of MPN-BP. Our findings indicate that MPN-BP arises by different molecular/cellular pathways present in individual patients that result in distinct clones/subclones that differ from patient to patient. These distinct clones/subclones coexist and emerge from different populations of MPN-BP IC.

## Results

### Somatic mutational patterns and karyotypic abnormalities present in MPN-AP/BP mononuclear cells (MNC) prior to treatment.

Capture-based next-generation sequencing (NGS) of MPN-AP/BP MNCs identified 2–7 mutations/patient (Pt), involving genes previously associated with myeloid malignancies (Supplemental Table 1; supplemental material available online with this article; https://doi.org/10.1172/jci.insight.156534DS1). *JAK2V617F* was the most common (61.1%), followed by mutations involving *TP53* (38.9%) and *MPL* (27.8%). Additional mutations were present, with the percentage of patients affected as follows: *TET2* and *ASXL1,* each at 22.2%; *RUNX1*, at 16.7%; *KRAS*, *SH2B3*, *SRSF2*, *SF3B1*, *U2AF1*, each at 11.1%; and *IDH1* at 5.6%. It is important to note that Pt 2 had a *TP53* mutation, which was accompanied by a deletion of chromosome 17, indicative of biallelic loss of p53 function. Cytogenetic analyses revealed that 12 (66.7%) patients at the time of their MPN-BP were cytogenetically normal, while the remaining 6 (33.3%) had multiple (2–7 abnormalities) chromosomal abnormalities coexisting in 1–2 clones. The 10 chromosomal abnormalities identified (+1q, –3, del[5q], –7, +8, –13, del[14q], –15, del[20q], –17) have been previously associated with intermediate- and adverse-risk acute myelogenous leukemia (AML) by the European LeukemiaNet (ELN) category (23). These findings indicate that MPN-AP/BP is characterized by a complex series of mutational and cytogenetic abnormalities that contribute to the biology of MPN-BP.

### MPN-BP SCs are capable of recapitulating human MPN-BP in NSG mice.

**Figure 1:**
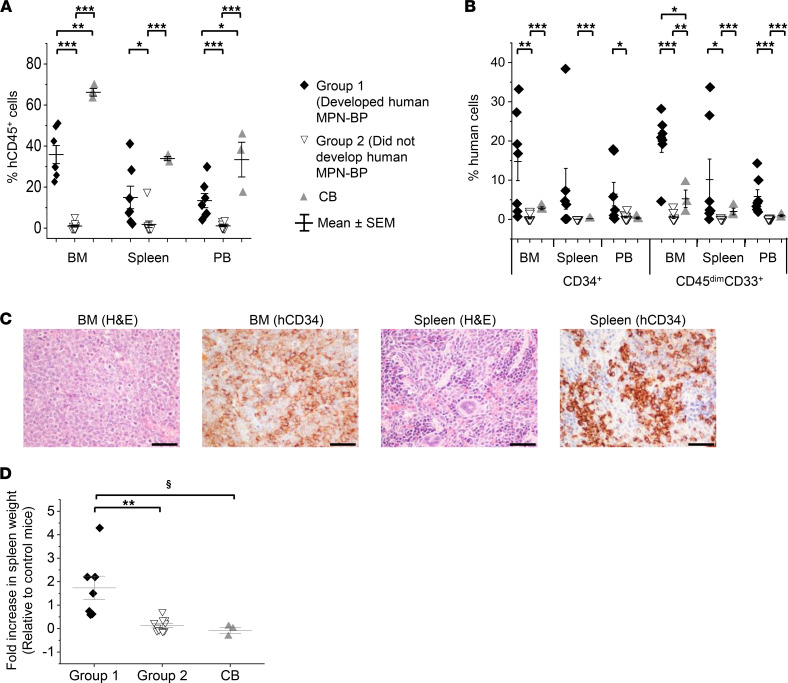
MPN-BP ICs are capable of engrafting and recapitulating MPN-BP in NSG mice. (**A** and **B**) Human CD45^+^ (hCD45^+^) cell chimerism (**A**) and the level of immature myeloid cells as indicated by percentage of hCD34^+^ and hCD45^dim^CD33^+^ cells in the BM, spleen, and PB of NSG mice receiving PB CD3^+^ cell–depleted MNCs from the 18 patients with MPN-AP/BP or normal CB CD34^+^ cells from 3 individual donors. Each symbol represents the mean from 2–4 different mice receiving one individual sample. (**C**) BM and spleen sections 8 weeks after the transplantation with primary samples from Pt 2 were stained with H&E and an anti-hCD34 mAb. Scale bars: 50 μm. (**D**) Fold increase in spleen weight of mice belonging to Group 1 and Group 2 relative to control mice. Control mice received PBS alone. For Group 1 (*n =* 7) samples, results were obtained from analyses of mice that were sacrificed 7 weeks (Pt 5), 8–9 weeks (Pts 2, 3, 4), and 16–18 weeks (Pts 1, 6, 7) after transplantation. For Group 2 (*n =* 11) samples, mice were sacrificed 4–7 months after the transplantation. *P < 0.05, **P < 0.01, ***P < 0.001, ^§^*P*=0.05 by ANOVA.

To determine whether MPN-BP ICs exist in the PB MNCs of patients with MPN-AP/BP, we performed xenograft repopulation assays. As shown in Supplemental Table 2, among the 18 patients studied, T cell–depleted MNCs from 13 patients established human cell chimerism in NOD-*scid* IL2Rg^null^ (NSG) mice. Samples from 7 patients (6 BP, 1 AP) resulted in 35.8% ± 7.3% marrow human CD45^+^ (hCD45^+^) cell chimerism and recapitulated numerous clinical features (3) of MPN-BP within 4 months of transplantation, including the presence of at least 20% hCD45^dim^CD33^+^ or hCD34^+^ cells (Supplemental Figure 1A) or at least 20% blasts, as detected by morphological examination of the BM and spleen. The 7 patient samples that were capable of recreating human MPN-BP in NSG mice were categorized as belonging to Group 1. By contrast, 5 patient samples (2 BP, 3 AP) achieved a lower degree of hCD45^+^ cell chimerism (Supplemental Table 2) and produced limited numbers of human myeloid blasts (hCD45^dim^CD33^+^, 1.1% ±0.7%; hCD34^+^, 0.8% ± 0.5%) in NSG mouse BM. The remaining 6 patient samples were not capable of engrafting NSG mice. The 11 samples that were not capable of causing human MPN-BP in NSG mice were categorized as belonging to Group 2. As shown in Figure 1, A and B, mice receiving Group 1 samples had significantly greater degrees of hCD45^+^ cell chimerism and leukemic cell burden, as indicated by the percent of hCD34^+^/hCD45^dim^CD33^+^ cells not only in the BM, but also in the spleen and PB, as compared with mice receiving Group 2 samples. Morphological and IHC analyses also revealed the presence of 50%–70% myeloid blasts in both the BM and the spleens of animals receiving grafts from a representative Group 1 patient (Pt 2) (Figure 1C), while Group 2 samples did not result in morphological changes that resembled MPN-BP (data not shown). The animals transplanted with Group 1 samples developed enlarged spleens and experienced a shortened lifespan (Figure 1D and Supplemental Table 2). Limiting dilution analysis revealed that the frequency of MPN-BP ICs in Group 1 samples was 107 times greater than that calculated for Group 2 samples (Supplemental Table 3). Moreover, as shown in Supplemental Figure 1, the degree of MPN-BP cell chimerism following transplantation of Group 1 samples was inversely related to the time to leukemia initiation and survival. The transplantation of normal cord blood (CB) cells exhibited a different engraftment pattern than that of MPN AP/BP cells. The degree of human cell chimerism achieved by CB cells was greater than Group 1 samples (Figure 1A); however, the BM xenografts were composed mostly of mature cells belonging to both myeloid and lymphoid lineages, with small fractions of myeloid blast cells (Figure 1B). These findings suggest that, unlike CB cells, human MPN-BP cells within the NSG mice were characterized by a myeloid maturation block that resembles MPN-BP. Our data, thus, clearly show that the MPN-BP ICs are present in MPN-AP/BP MNCs. We then focused our attention on a more detailed analysis of Group 1 samples, since the features of these xenografts resembled the clinical characteristics of human MPN-BP and since the high degree of chimerism that was achieved in these xenograft models allowed for further analysis.

### The behavior of MPN-BP ICs with serial transplantation.

**Figure 2:**
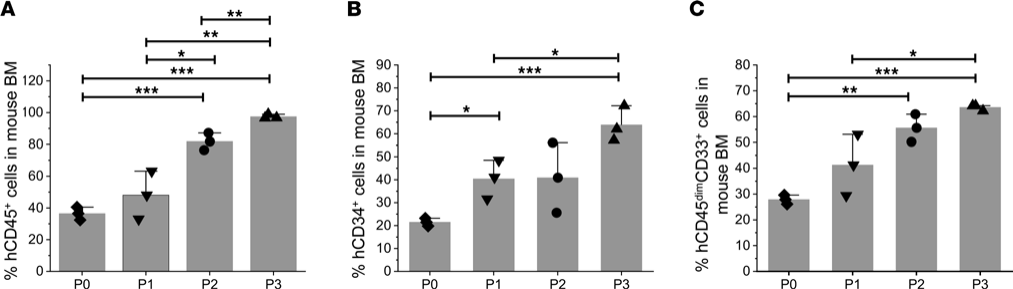
MPN-BP SC function following serial transplantation. (**A**–**C**) Human CD45^+^ cell chimerism (**A**) and leukemic cell burden (percentage of hCD34^+^ [**B**]/hCD45^dim^CD33^+^ cells [**C**]) were determined by flow cytometry of the BM of primary (P0), secondary (P1), tertiary (P2), and quaternary (P3) recipient mice receiving Pt 2 sample. Serially transplanted recipient mice were sacrificed 8 weeks after transplantation. Numbers of hCD34^+^ cells contained in the transplanted grafts are: Primary, 1.25 × 10^6^; P0, 4.6 × 10^5^; P1, 4.5 × 10^5^; P2, 2 × 10^5^. Bars indicate the mean in **A**–**C**. Data are represented as mean ± SEM. *n* each =3 mice. *P < 0.05, **P < 0.01, ***P < 0.001 by ANOVA.

We next determined the behavior of MPN-BP ICs following serial transplantation. Primary recipient mice are referred to as P0 recipients, and secondary, tertiary, and quaternary recipients are referred to as P1, P2, and P3 recipients, respectively. Among the 7 Group 1 individual specimens studied, 4 were capable of serially repopulating NSG mice and recreating MPN-BP after at least 1 passage (Figure 2 and Supplemental Figure 2, A–C), indicating that a fraction of MPN-BP ICs possessed properties characteristic of a true cancer SC. Moreover, the degree of human cell engraftment and leukemia cell burden progressively increased with subsequent serial transplantation (Figure 2 and Supplemental Figure 2, A–C). These data are especially striking, since the grafts that were serially transplanted were composed of far fewer numbers of hCD34^+^ cells than had been transplanted into the prior recipient (Figure 2 and Supplemental Figure 2, A–C). Moreover, the frequency of MPN-BP ICs increased with serial transplantation (Table 1 and Supplemental Table 4). The numbers of MPN-BP ICs after 1–3 passages were greater than the number of MPN-BP ICs present in the corresponding primary samples (Table 1 and Supplemental Table 4). These data indicate that serial transplantation of MPN-BP selected for a higher fraction of fully functional MPN-BP ICs. Furthermore, the OS of patients with MPN-BP cells (all treated with ruxolitinib and decitabine) that were capable of being serially transplanted was significantly shorter (2.0 ± 0.3 months) than that of the Group 2 patients whose MPN-AP/BP cells failed to recreate MPN-BP in NSG mice (9.0 ± 1.7 months) (Supplemental Figure 2D), suggesting that the behavior of these MPN-BP SCs in NSG mice correlated with the clinical outcomes of patients.

### Multiple distinct MPN-BP IC clones/subclones contribute to the development of MPN-BP.

**Figure 3:**
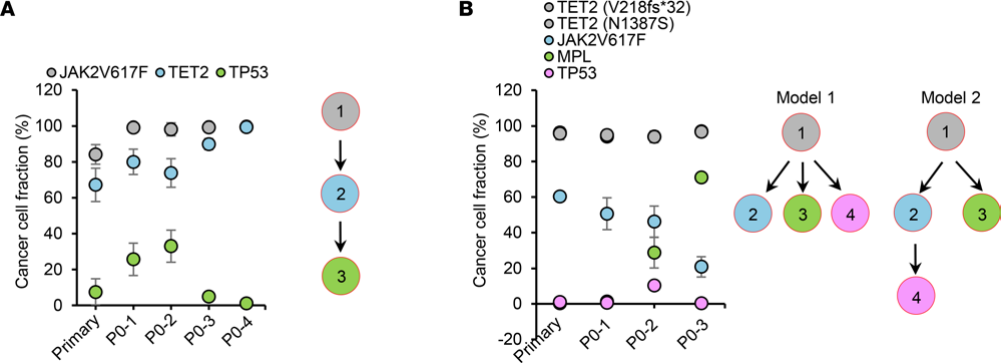
Multiple distinct MPN-BP IC clones/subclones coexist in patients with MPN-BP, which are capable of engrafting and recreating human MPN-BP in NSG mice. (**A** and **B**) The percentage of leukemic cells carrying each genetic mutation (cancer cell fraction [CCF]) in primary samples and in the corresponding individual P0 xenografts (left scatter plot) and the inferred MPN-BP IC clonal hierarchy (right diagram) for Pt 1 (**A**) and Pt 7 (**B**) belonging to Group 1. Mutations clustered together are indicated by the same color. Each circle represents a clone. Throughout each panel, the founder clone containing mutations indicated in dark gray in the left scatter plot is also shown in dark gray and is indicated in circle 1. Each subclone that is evolved from the parent clone is indicated by the circle of the same color that denotes the mutations newly acquired in the left scatter plot and is indicated by circles 1–4. In Pt 1, ClonEvol identified only 1 consensus model, while in Pt 7, this same program inferred 2 consensus models to explain the clonal hierarchy of MPN-BP ICs.

We next characterized the genomic architecture and karyotype of MPN-BP ICs. Human leukemia blasts present in the BM xenografts were selected by FACS (Supplemental Figure 3) and analyzed using NGS and FISH. As shown in Figure 3 and Supplemental Figure 4, primary xenografts resembled the mutational landscapes present in the corresponding patient’s primary samples; however, the presence of blast cells having the same genetic mutations in serially transplanted mice differed, enabling detection of MPN-BP IC subclones within the same sample. Cancer cell clones are defined as cancer cell populations containing unique sets of genetic aberrations. Thus, we were able to identify MPN-BP IC clones based on cancer cell fractions (CCF) for each mutation detected in the primary samples and human leukemia cells in NSG mice using the PyClone algorithm (Supplemental Table 5; ref. 24), and the inferred clonal hierarchy constructed using ClonEvol (25). As shown in Figure 3 and Supplemental Figure 4, MPN-BP founder clones underwent clonal evolution following either a linear and/or a branching evolutionary pattern, resulting in the generation of multiple subclones (2–3 subclones) containing several mutations with differing variant allele frequencies (VAF), which were identified in each of the primary samples, as well as corresponding xenografts. However, IC clonal competition also occurred following xenotransplantation, leading to a significant change in the myeloid gene mutations present in the xenografts as compared with the corresponding primary specimens. In addition, since libraries were sequenced with an average depth of 800× using a panel of 576 targeted genes associated with hematopoietic malignancies, clones that were present at a very low VAF (<2%) — and even clones that were not recognized in the original graft but emerged following xenotransplantation — were identified. A *TP53* oncogenic mutation (p.R175G) that was not present in Pt 7’s primary sample was detected in 1 of the 3 individual xenograft specimens (CCF, 51.8% ± 0.05%). Moreover, the chromosomal abnormalities present in the primary cells from each of the 5 patients belonging to Group 1 were also detected in leukemia cells within each paired xenograft (Table 2). Furthermore, some clones with cytogenetic abnormalities (–17 in Pt 2 and del[20q] in Pt 5; Table 2) exhibited a competitive proliferative advantage. These findings suggest that distinct MPN-BP IC subclones, as defined by their mutational burden and cytogenetic abnormalities, coexist in patients with MPN-BP and contribute to the development of MPN-BP.

### MPN-BP SC subclones differ in their proliferative and serial repopulating capacity.

**Figure 4:**
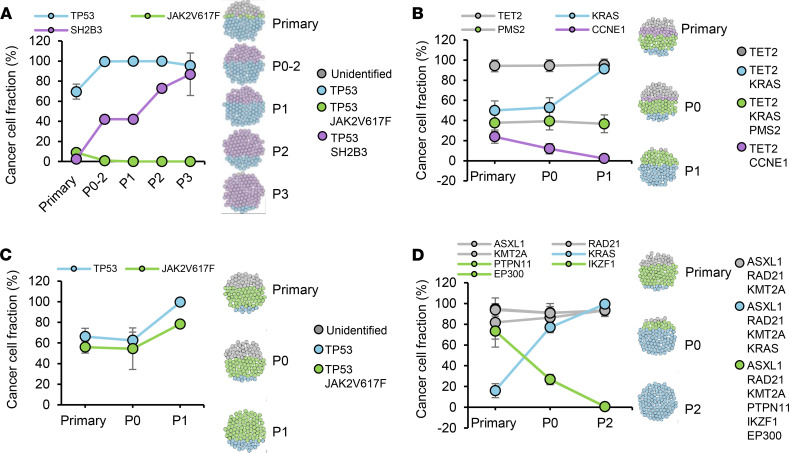
MPN-BP SC subclones differ in their proliferative and self-renewal capacity. (**A**–**D**) The percentage of leukemic cells with each genetic mutation (CCF) in primary samples from 4 patients and serial xenografts is shown in the left scatter plot, and clonal and subclonal populations within primary sample and serial xenografts are presented in the sphere of cells through each panel based on Model 1 of inferred clonal hierarchy if multiple consensus models are present in Figure 3 and Supplemental Figure 4. **A** shows Pt 2; **B** shows Pt 3; **C** shows Pt 4; **D** shows Pt 5. Primary MNCs from Pts 2 and 4 and sorted CD45^dim^CD33^+^ cells from Pts 3 and 5 were sequenced. For serial xenografts, hCD34^+^ cells derived from Pt 2’s primary sample and hCD45^dim^CD33^+^ cells derived from Pts 3–5 primary samples were sequenced.

In order to determine if some MPN-BP SC subclones have cell-intrinsic growth advantages that permit them to outcompete other subclones, we assessed the clonal composition of leukemia cells within serial xenografts from 4 patients with primary samples that were known to be capable of being serially transplanted. As shown in Figure 4 and Supplemental Figure 5, the clonal architecture of these patient samples changed dramatically following serial transplantation. Three major patterns of subclonal dynamics were observed: (a) Expansion was shown in that at least 1 genetically and 1 cytogenetically abnormal SC subclone in each patient had a competitive growth advantage that became progressively more predominant with serial transplantation. Moreover, in 2 of 4 patients, one or 2 minor subclones (CCF, 7.6%–16.1%, *SH2B3* [*LNK*] clone in Pt 2, *KRAS* clone in Pt 5) in the primary sample expanded and became the dominant or the exclusive clone present in the xenografts (Figure 4, A and D). (b) Stability was shown in that the CCF of 1 MPN-BP SC subclone containing *TET2*, *KRAS*, and *PMS2* mutations remained constant following serial transplantation (Figure 4B). (c) Loss was shown in that at least 1 genetically and/or 1 cytogenetically defined subclone in each patient was reduced in P0 or P1 recipients with most of these clones no longer detected in subsequent recipients (when available) (Figure 4, A, B, and D, and Supplemental Figure 5, A, C, and D), suggesting that these SC subclones possessed limited MPN-BP IC capacity. These observations indicate that several MPN-BP SC subclones that coexist in MPN-BP cells are undergoing continuous competition across multiple generations in the unrestrained environment of NSG mice. In addition, in all patients with an identifiable founder clone, the founder clone was progressively reduced or lost following serial transplantation (Figure 4, B and D), suggesting that the genetic mutations that define founder clones may initiate or even promote clonal expansion; however, additional genetic or cytogenetic alterations are needed to drive disease progression. These data provide evidence that MPN-BP SC subclones have distinct functional properties and that some possess cell-intrinsic proliferative properties, which likely contribute to the aggressive behavior of MPN-BP.

### Contributions of individual recurrent genetic mutations to the clonal expansion of MPN-BP ICs.

We next analyzed the consequence of *JAK2V617F* or mutations in *TP53*, *KRAS*, or *TET2*, which represented the most frequent mutations observed in MPN-BP ICs, by monitoring their VAFs after serial transplantation.

### JAK2V617F.

**Figure 5:**
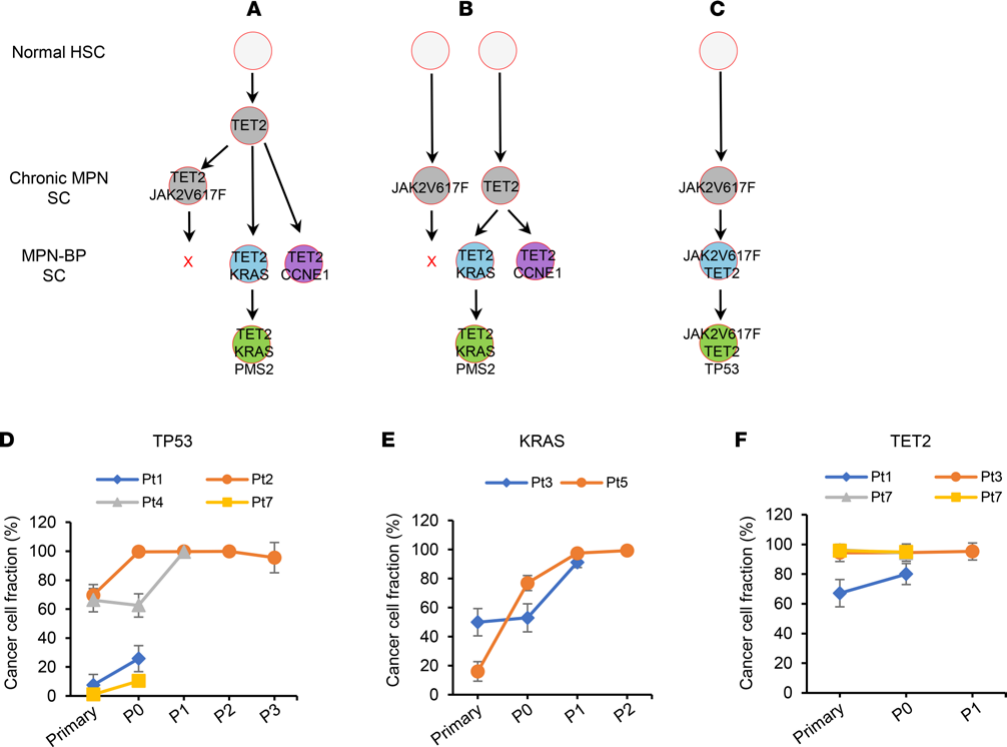
Contributions of recurrent genetic mutations to the clonal expansion of MPN-BP stem cells (SC). (**A**–**C**) Models explaining the relationship between chronic MPN and MPN-BP SCs. MPN-BP may arise from SCs distinct from the original chronic *JAK2V617F*^+^ MPN SCs (Pt 3, **A **and **B**) or directly from the *JAK2V617F^+^* MPN SCs as occurred in Pt 1 (**C**). (**D**–**F**) Mean percentage of leukemic cells (CCF) carrying mutations in *TP53* (**D**), *KRAS* (**E**), or *TET2* (**F**) genes in primary samples and serial xenografts generated from Group 1 samples. HSC, hematopoietic stem cell.

Unlike chronic myelogenous leukemia (CML), where BP evolves from the same clone from which CML originates, in 25%–41% of patients with a chronic *JAK2V617F*^+^ MPN that evolved to BP, the leukemic cells no longer possessed this driver mutation (15, 16). Similarly, in this report, the blast cells from 2 of 12 patients (Pts 3 and 5) with a *JAK2V617F*^+^ chronic MPN lacked this mutation when they evolved to MPN-BP (Supplemental Table 1 and Table 3). Moreover, *JAK2V617F* was not detected in either patient’s serial xenograft samples (Table 3). These observations suggest that MPN-BP may arise from distinct *JAK2V617F*^–^ SCs clonally related (Figure 5A) or unrelated to the MPN SCs (Figure 5B) (16). Within individual P0 xenografts derived from 2 patient samples (Pts 2 and 7), the *JAK2V617F* CCF was reduced (Table 3). Furthermore, the leukemia cells in the subsequent 3 serial recipients generated with Pt 2’s cells were *JAK2* WT (Table 3). Although the CCF of *JAKV617F^+^* leukemia cells was increased in P0/P1 xenografts derived from the other 2 patients (Pts 1 and 4) (Table 3), *JAKV617F* alone did not confer a proliferative advantage to MPN-BP ICs, but rather other coexisting mutations such as *TP53* (Pt 1, Supplemental Figure 6A; Pt 4, Figure 4C) were responsible for the expansion of *JAKV617F^+^* MPN-BP IC. These findings suggest that, in a subset of patients, *JAK2V617F* may contribute to the progression of the chronic MPN to BP (Figure 5C) but that MPN-BP IC activity is not always dependent on *JAK2V617F*.

### TP53 and KRAS mutations.

The CCF for *TP53* and *KRAS* mutations increased in paired P0/P1 xenografts from all patient samples that had either mutation, even where a small fraction of *TP53-* or *KRAS*-mutated cells was present (*TP53*, Pts 1 and 7; *KRAS*, Pt 5) (Figure 5, D and E). Moreover, *TP53* mutations served as the driver mutation of a subclone in 3 of 4 patients with *TP53* mutations (Figure 4C and Supplemental Figure 6), and *KRAS* mutations were the driver mutation in subclones that experienced clonal expansion in both patients with this type of mutation (Figure 4, B and D). These observations support the hypothesis that acquisition of *TP53* or *KRAS* mutations provides MPN-BP ICs with a proliferative advantage, irrespective of the size of the mutant subclone present in the primary cells.

### TET2 mutations.

Three distinct *TET2* mutations were present as heterozygous founder clonal mutations in the primary cells of 2 patients, Pts 3 and 7 (Figure 5F), indicating that *TET2* mutations may represent early events but that additional genetic mutations are required to transform a chronic MPN to BP, such as *KRAS* mutation in Pt 3 (Figure 4B) and *JAK2V617F*, *MPL*, and *TP53* mutations in Pt 7 (Figure 3B).

### MPN-BP ICs originate from HSCs and/or HPCs.

**Figure 6:**
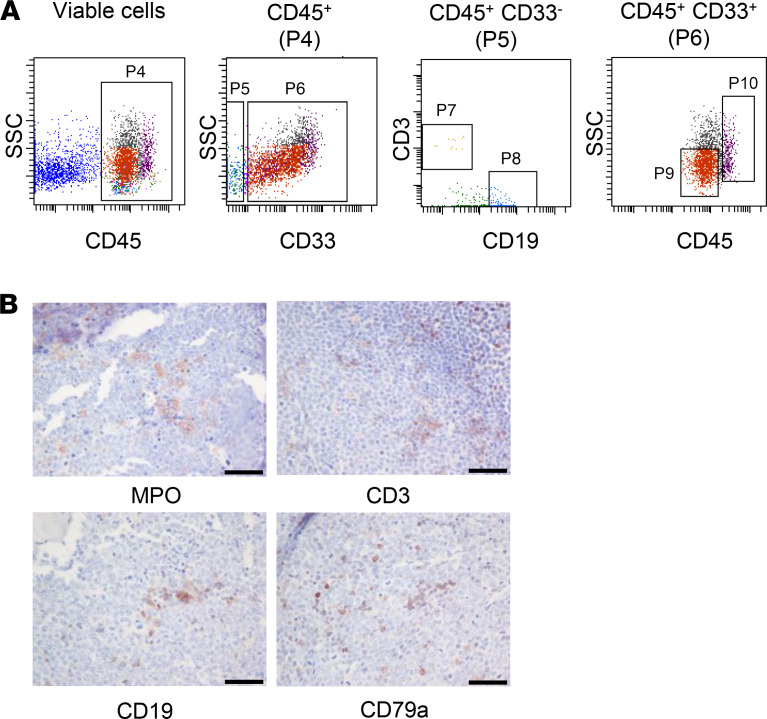
MPN-BP SCs retain the capacity to differentiate into mature myeloid and lymphoid cells. (**A**) FACS plots showing multilineage differentiation capacity of a representative primary sample from Pt 2 in P0 recipient mice. (**B**) Immunohistochemical analysis of the BM of NSG recipient mice. Multilineage hematopoietic cell differentiation of a representative sample from Pt 2 in NSG recipient mice is shown. Scale bars: 50 μm. MPO, myeloperoxidase.

**Figure 7:**
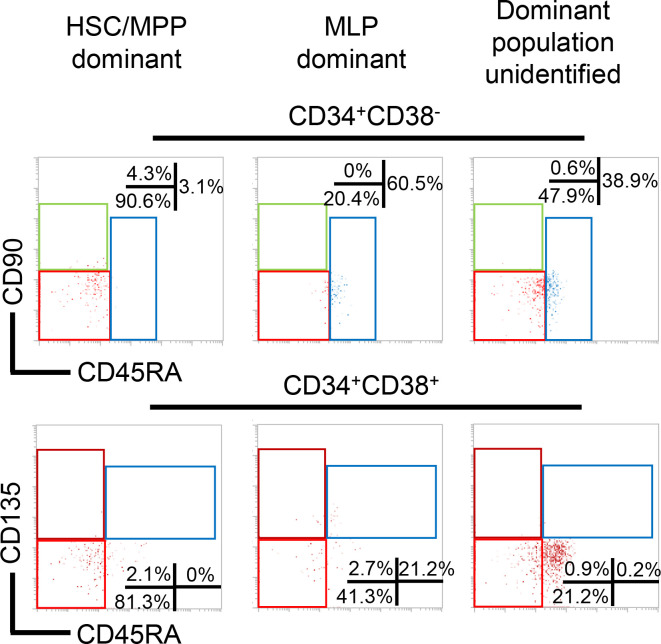
Phenotypic analysis of HSC/HPC subpopulations in MPN-BP primary samples. CD90 and CD45RA expression in CD34^+^CD38^–^ cells (upper panel) and CD135 and CD45RA expression in CD34^+^CD38^+^ cells (lower panel) from a representative MPN-BP sample where HSC/MPP population was dominant (left), a representative MPN-BP sample where MLP population was dominant (middle), and a representative MPN-BP sample where HSC/MPP and MLP populations were distributed equally. HSC, hematopoietic stem cell; MPP, multipotent progenitor; MLP, multilymphoid progenitor.

In order to identify the cell of origin of MPN-BP ICs, we initially examined their lineage differentiation potential. Flow cytometric analysis revealed that MPN-BP ICs present in all the 7 Group 1 primary samples displayed skewed differentiation toward the myeloid lineage. However, smaller numbers of lymphoid cells were generated by 6 samples (Figure 6A and Table 4). The myeloid cells within the xenografts were composed primarily of myeloid blasts (CD45^dim^CD33^+^) with a small fraction of mature myeloid cells (MMC) (CD45^int/bright^CD33^+^) (Figure 6A), CD14^+^ monocytes, and CD41a^+^ megakaryocytes (data not shown). IHC analyses confirmed this lineage distribution within a xenograft (Figure 6B). Surprisingly, the patient-specific genetic and chromosomal abnormalities either were present at similar frequency in myeloid blasts, MMC, and lymphocytes within serial xenografts generated from primary cells from Pts 1–3 (Table 5 and Supplemental Figure 7, A and B) or were occasionally differentially represented in cells belonging to specific cell lineages (Pt 4, Supplemental Figure 7C; Pt 5, Supplemental Table 6). These data indicate that most MPN-BP IC subclones are still capable of differentiating into MMC and lymphocytes, suggesting that they likely originate at the level of HSC/multipotent progenitor (MPP) or multilymphoid progenitor (MLP). We then analyzed the distribution of the various HSC/HPC subpopulations based on their respective phenotypes in primary MPN-BP cells from 21 patients. As shown in Figure 7 and Table 6, in 23.8% of cases, an MLP phenotype was dominant, while in 61.9% of cases, HSCs/MPPs predominated. In the remaining 3 samples, the numbers of HSCs/MPPs and MLPs were equally distributed. Within the CD34^+^CD38^+^ cell compartment, the MEP population predominated in 18 of 21 MPN-BP samples. The identification of various HSC/HPC subpopulations in primary MPN-BP cells, therefore, allowed us to isolate specific cell populations along the hematopoietic developmental hierarchy to determine the cell types with MPN-BP IC activity.

First, NGS and FISH revealed that sorted HSCs/MPPs and MLPs from Pt 2 and Pt 5 — whose bulk samples had previously been shown to be capable of being serially transplanted and to create MPN-BP in NSG mice — as well as mixed myeloid committed progenitors (MMCP, common myeloid progenitors [CMP] + MEP + granulocyte-monocyte progenitors [GMP]) isolated from Pt 2 (Supplemental Figure 8) — contained almost all the mutations and chromosomal abnormalities identified in the corresponding primary cells, as well as the blasts isolated from xenografts (Table 5 and Supplemental Table 6). The MPN-BP dominant chromosomal abnormality –17 was present in primary T cells isolated from Pt 2 at a similar level as in primary blasts and primary HSCs/MPPs (Table 5). These data indicate that MPN-BP IC activity is shared by multiple types of primitive hematopoietic cells, as defined by phenotype.

**Figure 8:**
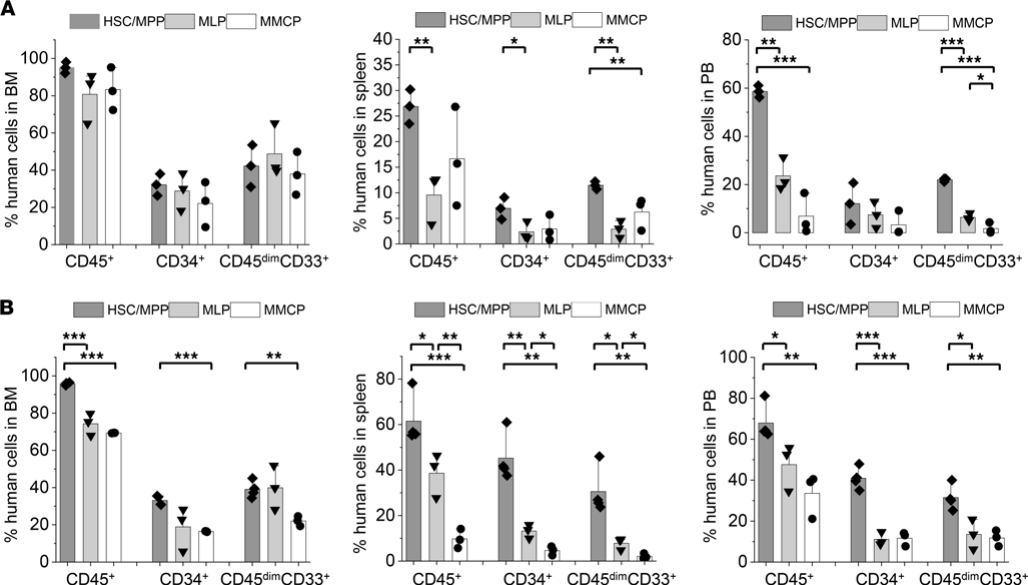
MPN-BP ICs originate from multiple HSC/HPC compartments. (**A**) Human cell engraftment (% hCD45^+^ cells) and leukemic burden (% hCD45^dim^CD33^+^ or % hCD45^+^CD34^+^ cells) in the BM (left), spleens (middle), and PB (right) of P0 recipients 8–9 weeks after the transplantation of equal numbers of HSCs/MPPs, MLPs, and mixed myeloid committed progenitors (MMCP, CMP + MEP + GMP) sorted by FACS from Pt 2 PB sample. *n* each=3 mice. (**B**) Human cell engraftment and leukemic burden in the BM (left), spleens (middle), and PB (right) of P1 recipients 6–7 weeks after the transplantation of BMCs collected from P0 recipients receiving primary HSCs/MPPs, MLPs, or MMCPs from Pt 2. HSC/MPP, *n =* 4 mice; MLP and MMCP, *n =* 3 mice. Bars indicate the mean in **A** and **B**. Data are shown as mean ± SEM. *P < 0.05, **P < 0.01, *** *P* < 0.001 by ANOVA. Please note similar patterns of leukemia cell engraftment and MPN-BP development were observed when BMCs from P1 recipients were transplanted into P2 recipients (data not shown).

**Figure 9:**
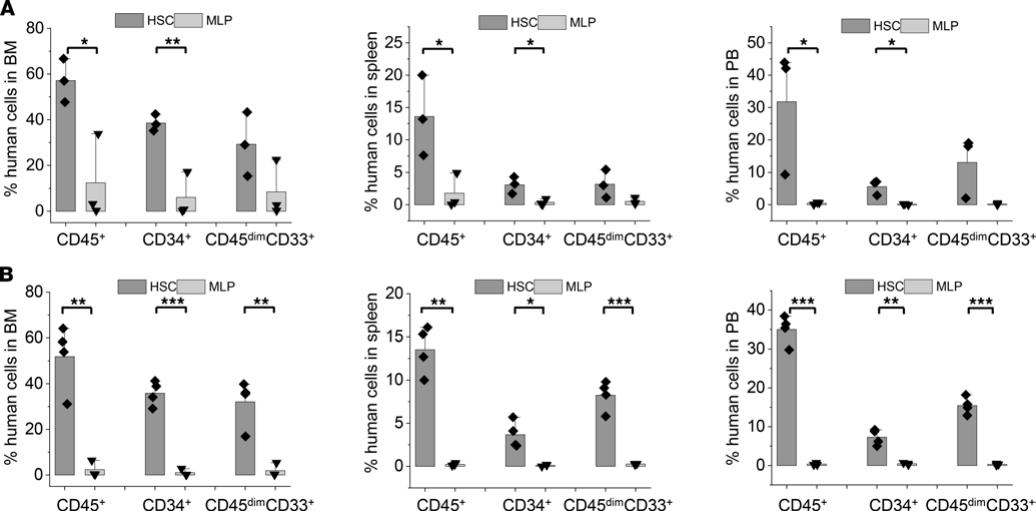
MPN-BP ICs originate from both the HSCs and MLPs within the MNCs of Pt 5. (**A**) Human cell engraftment (% hCD45^+^ cells) and leukemic burden (% hCD45^dim^CD33^+^ or % hCD34^+^ cells) in the BM (left), spleens (middle), and PB (right) of P0 recipients 11–12 weeks after the transplantation of equal numbers of HSCs and MLPs sorted by FACS from Pt 5 PB sample. *n* each=3 mice. (**B**) Human cell engraftment (% hCD45^+^ cells) and leukemia cell burden (% hCD45^dim^CD33^+^ or % hCD34^+^ cells) in the BM (left), spleen (middle), and PB (right) of P1 recipients 9–10 weeks after the transplantation of BMCs collected from P0 recipients receiving purified HSCs or MLPs from Pt 5. HSC, *n =* 4 mice; MLP, *n =* 3 mice. Bars indicate the mean in **A** and **B**. Data are shown as mean ± SEM. *P < 0.05, **P < 0.01, *** *P* < 0.001 by ANOVA. Please note HSC grafts were capable of engrafting and generating a similar high level of leukemia cell burden in P2 recipients, as observed in P1 recipients. By contrast, MLPs were not capable of engrafting P2 recipients (data not shown).

Next, we assessed if any or all of the HSC/HPC populations purified from these 2 patients had MPN-BP IC activity. The MPN-BP IC activity of the MEP population alone could not be evaluated, since limited numbers of these purified cells were isolated from Pt 2. To determine if myeloid-committed progenitors present in CD34^+^CD38^+^ cells had MPN-BP IC activity, CMPs, MEPs, and GMPs from Pt 2 were pooled (MMCP) and transplanted into NSG mice. As shown in Figure 8, HSC/MPP, MLP, and MMCP populations from Pt 2 each possessed MPN-BP IC activity and self-renewal capacity, but HSCs/MPPs had a greater proliferative potential than the other 2 cell populations. Each of these 3 primary cell populations showed skewed myeloid differentiation, although a very small number of lymphoid cells was generated by HSCs/MPPs and MLPs (Table 7). Moreover, the blasts derived from each of these populations contained the *TP53* mutation and –17, and the B cells contained the dominant chromosomal abnormality –17 (Table 8). Furthermore, in Pt 5, only cells expressing the HSC phenotype were capable of recreating human MPN-BP in NSG mice and were capable of engrafting P2 recipients, whereas MLP cells had limited proliferative capacity and self-renewal potential (Figure 9, Supplemental Table 7, and Supplemental Table 8). These data suggest that MPN-BP SCs originate from not only phenotypically identified HSCs, but also lymphoid-myeloid progenitor cells, which differ in MPN-BP initiating activity and self-renewal capacity. In addition, the transplantation of HSCs/MPPs from both patients was associated with a more fulminant form of MPN-BP, evidenced by a shorter survival of the P0/P1 recipients (Supplemental Figure 9, A–D), as compared with mice transplanted with MLPs or MMCPs.

## Discussion

In this study, we used an MPN-BP serial NSG mouse xenograft system to further explore the properties of MPN-BP ICs. The transplantation of samples from 18 different MPN-AP/BP patients allowed us to define 2 groups of MPN-BP based on their ability to engraft NSG mice. Although engrafting Group 2 samples achieved limited hCD45^+^ cell chimerism in the BM of primary recipients, the BM xenografts were composed of the same percentage of myeloid blasts as that observed with Group 1 samples (hCD45^dim^CD33^+^/hCD45^+^ cells: Group 1, 55.4% ± 11.7%; Group 2, 55.0% ± 15.7%). Moreover, we were able to isolate sufficient hCD45^+^ cells from the BM of 1 mouse that had been transplanted with a Group 2 sample to perform NGS and found that the human cells had the identical clonal architecture as that of the primary sample (Supplemental Table 9). These findings suggest that MPN-BP ICs present in Group 2 samples were leukemic in nature but had a limited capacity to engraft and/or proliferate in the hematopoietic microenvironment present within NSG mice. The distinct growth properties observed by Group 1 and Group 2 MPN-BP ICs in NSG mice might be due to either intrinsic biological features that distinguish these 2 groups of samples or the inability of NSG mouse BM microenvironment to support the development of MPN AP/BP from Group 2 MPN-BP ICs.

Previous studies of acute lymphoblastic leukemia (ALL) and colorectal cancer have demonstrated that tumor subclones can display enhanced engraftment potential following serial passaging in NSG mice (26–30). Recently, Sandén et al. have demonstrated that serial transplantation over longer periods of time exposes inherent differences in the proliferation and self-renewal capacity of competing AML clones and allows modeling of clonal dynamics in vivo (31). In this study, we have focused on the clonal dynamics of functional MPN-BP SC subclones with different mutations and cytogenetic abnormalities that were capable of engrafting and recreating MPN-BP in NSG mice. The MPN-BP SC subclonal dynamics were consistent with MPN-BP SC functional evolution and disease progression observed in serial xenografts. These observations made from studying primary patient samples having a wide number of initiating genetic events have important clinical implications. Although molecularly targeted therapies have led to clinical remissions and clonal responses in some cancer patients (32), relapse frequently occurs and is often associated with evidence of clonal evolution, most likely reflecting intratumoral heterogeneity, which is present at diagnosis (33). In approximately 50% of patients with ALL and AML, the clone responsible for relapse is not related to the predominant clone present at diagnosis, but rather to minor leukemic subclones (34, 35). MPN-BP SC subclonal dynamics, as observed here (Figure 4), suggests that minor MPN-BP SC subclones in most cases may acquire an increased proliferative potential following serial transplantation. Due to the limited efficacy of the therapies currently used to treat patients with MPN-BP, we conjecture that effective therapy for MPN-BP should optimally be designed to eradicate each of the subclones present within MPN-BP MNCs. We speculate that the subclones that are not apparent in primary cells but can be detected in NSG mice might eventually lead to a relapse after the administration of more effective chemotherapy.

The dynamics of genetic and cytogenetic abnormalities in primary samples and xenografts not only enabled us to identify functional MPN-BP SC clones/subclones and to assess the clonal relationship between the chronic MPN and MPN-BP, but it also allowed us to assess the contribution of *JAK2V617F* and other recurrent mutations to LT and/or clonal expansion of MPN-BP ICs. Our studies (Table 3, Figure 4C, and Supplemental Figure 6A) and those of others (15, 36) suggest that *JAK2V617F* alone does not confer a growth advantage to MPN-BP IC clones/subclones. These data may provide an explanation for the loss or decrease of *JAK2V617F* in a subset of patients at the time of MPN-BP. Prior xenograft and mouse model studies have already indicated that HSCs harboring *JAK2* mutations have limited or even lack a competitive survival/proliferative advantage relative to normal HSCs (37–41). Although *JAK2V617F* may not directly initiate LT or drive MPN-BP SC clonal expansion in a subset of patients with MPN-BP, we speculate that *JAK2V617F* may still promote MPN-BP development and further disease progression by creating an inflammatory milieu that contributes to disease progression (42–49). Unlike *JAK2V617F*, in 5 patients who possessed *MPL* mutations, these mutations were not lost but persisted or were acquired at the time of MPN-BP. Moreover, the xenografts generated with samples from those individuals with *MPL* mutations were characterized by the persistence of *MPL* mutations (Pt 6, Supplemental Figure 4E; Pt 7, Figure 3B). In some situations, the *MPL* mutation persisted in the xenograft cells with *JAK2V617F* (Pt 7). These observations suggest that the TPO/MPL axis might play a role in the origins of MPN-BP. MPL is a type 1 homodimeric cytokine receptor that has been proposed to play a pivotal role in the development of chronic MPNs (50, 51). Further clarification of the role of the TPO/MPL axis in the development of MPN-BP will require more extensive studies of larger patient populations. In addition, our findings establish a clear association between *TP53* and *KRAS* mutations with aggressive behavior of MPN-BP ICs and the dismal outcomes of patients who possess these 2 types of mutation, as has been suggested by others (52). Mutations in other genes such as *ASXL1*, *EZH2*, *SRSF2*, and *SH2B3* were also more frequently observed in primary samples of patients who had experienced transformation of MPN into BP (1, 2, 7–13), suggesting a role for these events in LT. However, to what extent these genomic events contribute to MPN-BP clonal expansion and disease progression remains to be elucidated.

MPN-BP has a unique mutational pattern and molecular pathogenesis that is distinct from de novo AML (7–13, 53–56). *JAK2V617F* is the most common event in MPN-BP but is not observed in de novo AML (7, 53), whereas mutations in *FLT3*, *NPM1*, *DNMT3A*, which are most common events in de novo AML, are rarely observed in MPN-BP (7). Moreover, most cases of de novo CD34^+^ AML originate from phenotypically identifiable HPC, including MLP and/or GMP, which acquire abnormal self-renewal potential; this suggests that CD34^+^ AML is a progenitor cell disease (18). Recently, studies of de novo AML have identified the presence of preleukemic HSCs that are ancestral to the dominant AML clone, which originates from the MLP compartment. These preleukemic HSCs harbor mutations associated with myelodysplasia (e.g., of *DNMT3A*, *TET2*, and others), which persist in myeloid cells as well as B and T cells but lack the mutations present in leukemic blasts (57–60). By contrast, in MPN-BP, not only HSCs/MPPs, but also MLPs, exhibited MPN-BP IC activity. However, MPN-BP ICs present in the HSC/MPP compartment had a greater proliferative capacity and serial repopulating potential than those originating from MLP or myeloid committed progenitor compartment. These observations suggest that MPN-BP is a HSC/MPP disease and that MPN-BP MLPs may participate but possess more limited MPN-BP IC activity. Ho et al. have reported that several years prior to the diagnosis of BP, BP-specific myeloid leukemia gene mutations were detected within HSC populations from several CP MPN patients, supporting the HSC origin of BP (61). Our studies provide evidence that MPN-BP has a different cell of origin than de novo AML, which might account for inherent biological differences between these types of acute leukemias beyond their distinctly different mutational patterns.

In conclusion, our studies indicate that MPN-BP ICs display extensive clonal, functional, and phenotypical heterogeneity within an individual patient and that MPN-BP is a distinct disease from de novo AML; these conclusions are likely responsible for the limited effectiveness of presently utilized therapeutic approaches. Additional systematic studies of the molecular and cellular characteristics of MPN-BP ICs might, therefore, lead to the development of novel targeted therapeutic strategies capable of eliminating MPN-BP ICs.

## Methods

### Animals.

NSG mice were purchased from The Jackson Laboratory. NSG and PDX mice were housed in the ISMMS animal facility.

### Patient specimens.

Baseline PB samples were collected from patients with MPN-AP/BP prior to their being treated with ruxolitinib and decitabine in a Phase I/II trial (NCT02076191) (6). This trial was sponsored by the MPN-Research Consortium (MPN-RC). The genetic and cytogenetic features of 18 patients are shown in Table 1.

### Cells.

MNCs were isolated by density gradient centrifugation using Ficoll-Paque (GE Healthcare Life Sciences). CD3^+^ cells were depleted from PB MNCs using an EasySep human CD3^+^ selection kit (Stemcell Technologies). Flow cytometric analyses confirmed that < 0.02% CD3^+^ cells remained in the depleted MNCs using a FACSCanto Flow Cytometer (BD Biosciences). CB CD34^+^ cells were selected from CB MNCs using an EasySep human CD34^+^ selection kit II (Stemcell Technologies). CB CD34^+^ cells with a purity of ≥ 95% were used in this study.

### Xenograft repopulation assay.

CB CD34^+^ cells (5 × 10^5^/mouse, *n =* 3) or PB CD3^+^ cell–depleted MNCs from the 18 patients were transplanted via the tail vein of sublethally irradiated (220–240 cGy) 8- to 9-week-old NSG mice either using a serial diluting approach (1 × 10^2^ to 1 × 10^7^ MNCs/mouse, 3–5 mice/group) or at a fixed cell number (1.5 × 10^6^ to 10 × 10^6^ MNCs/mouse) depending on the number of cells available. After transplantation, mice that had ≥ 3% leukemic cell chimerism (hCD45^dim^CD33^+^ or hCD34^+^ cells) in PB and had greater than 20% body weight loss were sacrificed 8–9 or 16–18 weeks after transplantation, unless otherwise indicated. Mice transplanted with samples that had a normal performance status and had normal blood counts were sacrificed 4–7 months after transplantation. After the mice were sacrificed, cells were recovered from the BM, spleen, and PB of recipient mice. The presence of human hematopoietic lineage cells was determined by mAb staining and flow cytometric analysis. The source of these mAbs is provided in Supplemental Table 10. Human engraftment was considered to have occurred if hCD45^+^ cells were present at ≥ 0.1% in the BM or spleen of a recipient mouse.

For morphological and IHC evaluation, sections were obtained from formalin-fixed, decalcified, paraffin-embedded femurs and spleens dissected from the NSG mice transplanted with MPN-AP/BP cells. These sections were stained with H&E and Ab specific for hCD34, hCD279a, hCD3, and hCD19, as well as myeloperoxidase (MPO), to further evaluate human leukemia cell morphology and the human hematopoietic cell lineages represented. The source of these antibodies is provided in Supplemental Table 10. Following appropriate antigen retrieval, IHC stains were performed using an automated immunostainer (Bond Max). 

In order to examine the serial transplantability of MPN-BP SCs, BM cells harvested from primary recipient mice were transplanted into secondary followed by tertiary and quaternary recipients in a similar fashion as described above either using a serial dilution approach (1.0 × 10^1^ to 1.0 × 10^6^, 3–5 mice/group) or with a fixed number of donor cells (0.5 × 10^6^ to 2.5 × 10^6^/mouse). BM cells from the same mouse that had been transplanted into subsequent recipient mice were analyzed by NGS and FISH, as described below.

In order to assess which HSC/HPC population had MPN-BP IC activity, HSC/MPPs, MLPs, and MMCPs sorted by FACS from Pt 2 primary sample, as well as HSCs and MLPs from Pt 5 primary sample (1 × 10^5^ cells/mouse), were transplanted into 4–6 NSG mice in the same fashion as the bulk primary sample. To determine if any cell population had self-renewal capacity, BM cells (containing 1 × 10^5^ hCD34^+^ cells) collected from P0 recipients were transplanted into P1 NSG mice (*n =* 6–7).

### Capture-based NGS.

NGS was performed using a targeted sequencing panel, including 576 genes associated with hematologic malignancies, as previously described (62). Libraries were sequenced on an Illumina HiSeq 2500 with 2 × 125 bp paired-end reads with an average depth of 800×. Sequencing reads were aligned to human genome (hg19) using BWA-MEM algorithm (63) (v. 0.7.12-r1039), and the data quality was assessed using FastQC (http://www.bioinformatics.babraham.ac.uk/projects/fastqc/).

### Identification of substitutions and small insertion/deletions.

Mutations were called using CAVEMAN (64) (Ver. 1.7.4), Mutect (65) (Ver. 4.0.1.2), Strelka (66) (Ver. 2.9.1), and PINDEL (67) (Ver. 1.5.4) and were subsequently annotated with Ensembl Variant Effect Predictor (68) (Ver. 86) and OncoKb (69). A subset of all candidate mutations that passed confidence criteria or matched a known somatic mutation was retained for manual review. The variants presented in this study are those that were identified as pathogenic or likely pathogenic.

The mutational pattern of the sorted primary stem/progenitor cell populations and primary leukemic blasts from Pt 2 — as well as sorted human leukemic blasts, MMC, T cells, and B cells from serial xenografts, if available, derived from Pts 1–7 — were analyzed in a similar fashion. Due to the limited number of primary stem/progenitor cell populations and leukemic blasts available from Pt 5, whole genome application of these cell populations was performed before NGS. For primary samples, MNCs from Pts 1, 2, 3, 4, 5, 6, 7, which contained 91.6%, 83.5%, 98.7%, 95.2%, 93.4%, 82.2%, and 94.3% myeloid blasts, respectively, and sorted CD45^dim^CD33^+^ cells from Pts 2, 3, 5 were sequenced. For xenografts, sorted hCD45^dim^CD33^+^ cells (Pts 1 and 3–7) and hCD34^+^ cells (Pt 2) were sequenced. The mutations and their VAFs in each individual primary and xenograft sample are shown in Supplemental Table 11.

The accession no. for the NGS data sets reported in this paper is SRA: PRJNA691948 (https://submit.ncbi.nlm.nih.gov/subs/sra/SUB8756235/overview).

### Copy number analysis.

The CNACS (70) algorithm was used to assess copy number alterations based on NGS sequencing data. This algorithm is optimized for targeted assays and uses a panel of normals for allele-specific detection of copy number changes, as well as regions of copy number neutral loss of heterozygosity (cnLOH).

### Karyotypic analyses and FISH studies to detect chromosomal abnormalities.

Unstimulated G-banded metaphases of primary PB MNCs from these 18 patients were obtained using standard methodology (71). ISCN chromosome nomenclature was used to describe chromosomal abnormalities (72). Primary MNCs from Pts 1 and 4; selected primary leukemic blasts from Pts 2, 3, and 5; primary T cells from Pt 2; and selected human leukemic blasts, MMC, T cells, and/or B cells if available from xenografts derived from 5 patients belonging to Group 1 were evaluated for the presence of indicated chromosomal abnormalities contained in primary samples using interphase FISH, as previously described with pepsin modification treatment (100 μL 10% pepsin and 2 mL 1% HCl) for 5 minutes (71). About 10–100 nuclei were scored in each of the cell populations. The source of FISH probes is provided in Supplemental Table 10.

### Clonal analysis.

The analysis of the clonal composition of a particular patient with MPN-AP/BP was based on the appearance of recurrent myeloid gene mutations using the PyClone algorithm (24). First, the CCF (the percentage of leukemic cells with each mutation) in primary patient samples and their corresponding xenografts was calculated by adjusting the VAF for ploidy, based on the copy number analysis. PyClone-generated clusters in Group 1 primary samples and the corresponding xenografts are presented in Supplemental Table 5. The clonal hierarchy was inferred, and clonal and subclonal populations within a sample were visualized using ClonEvol (25).

### FACS.

MNCs (1 × 10^6^ in 100 μL) from the PB of Pts 2 and 5 were stained with the following mAbs: anti–CD33-PerCP-Cy5.5, anti–CD34-APC, anti–CD45-FITC, anti–CD38-APC-Cy7, anti–CD45-RA-PE-Cy7, anti–CD90-PE, anti–CD7-BV-510, anti–CD10-PE-Cy5, anti–CD135 PE, and anti–CD3-FITC. Nonleukemic HSC/HPC populations — including HSC/MPP, MLP, CMP, GMP, and MEP within the CD33^–^ cell fraction, as well as CD45^dim^CD33^+^ leukemic blasts from Pts 2 and 5 — were isolated using an IMI5L cell sorter (BD Biosciences) to a postsort purity of > 95% (Supplemental Figure 8). The phenotypes of these cell populations were as follows: HSC, CD34^+^CD38^–^CD90^+^CD45RA^–^; MPP, CD34^+^CD38^–^CD90^–^CD45RA^–^; MLP, CD34^+^CD38^–^CD90^+/–^CD45RA^+^; CMP, CD34^+^CD38^+^CD7^–^CD10^–^CD135^+^CD45RA^–^; GMP, CD34^+^CD38^+^CD7^–^CD10^–^CD135^+^CD45RA^+^; and MEP, CD34^+^CD38^+^CD7^–^CD10^–^CD135^–^CD45RA^–^ (57). Human leukemic blasts (hCD45^dim^CD33^+^/CD34^+^), MMC (hCD45^int/bright^CD33^+^/CD14^+^), and T (hCD45^+^CD33^–^CD3^+^) and/or B (hCD45^+^CD33^–^CD19^+^) cells when present in sufficient numbers were also selected from the BM of NSG mice transplanted with human MPN-BP cells from 7 patients belonging to Group 1 (Supplemental Figure 3). The source of these mAbs is provided in Supplemental Table 10.

### Statistics.

Statistical analysis was performed by 1-way ANOVA using Origin 2021b software for Windows (http://www.originlab.com). *P* values less than 0.05 were considered significant unless otherwise specified. The limiting-dilution analyses of MPN-BP ICs were performed using Poisson statistics with L-Calc software (STEMCELL Technologies).

### Study approval.

All patients provided written informed consent under a protocol approved by the IRB of ISMMS or participating organizations in the MPN-RC. All animal experiments were approved by the Animal Care Committee of the ISMMS and were performed following the NIH guidelines (*Guide for the Care and Use of Laboratory Animals,* National Academies Press, 2011).

## Author contributions

XW and RH conceived the study; XW designed the study, performed experiments, analyzed and interpreted data, and wrote and revised the manuscript; CSH performed experiments and analyzed data; JT and VN performed cytogenetic analysis; RKR, NF, MRR, MP, and EM provided NGS data; BP interpreted histopathology and IHC; MK and RSW performed sample procurement and curation; RH interpreted data and revised the manuscript for first submission; and XW, JT, VN, RKR, BP, CIR, ML, AD, JM, and RH reviewed and edited the manuscript. All the authors contributed to the article and approved the submitted version.

## Supplementary Material

Supplemental data

Supplemental table 5

Supplemental table 11

## Figures and Tables

**Table 8:**
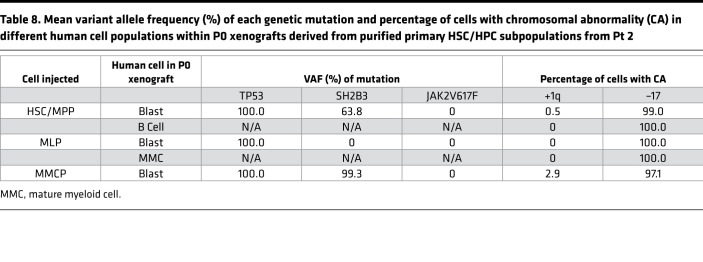
Mean variant allele frequency (%) of each genetic mutation and percentage of cells with chromosomal abnormality (CA) in different human cell populations within P0 xenografts derived from purified primary HSC/HPC subpopulations from Pt 2

**Table 7:**
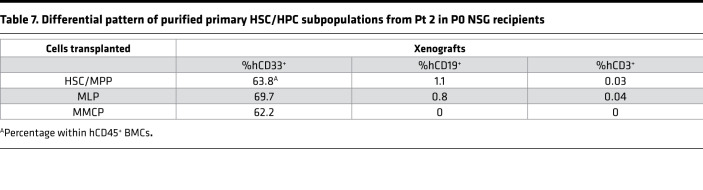
Differential pattern of purified primary HSC/HPC subpopulations from Pt 2 in P0 NSG recipients

**Table 6:**
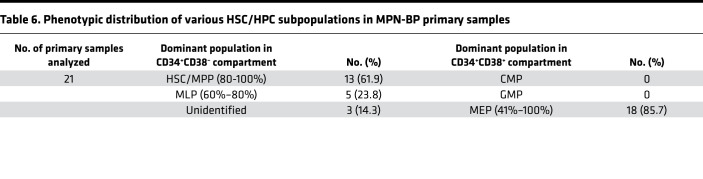
Phenotypic distribution of various HSC/HPC subpopulations in MPN-BP primary samples

**Table 5:**
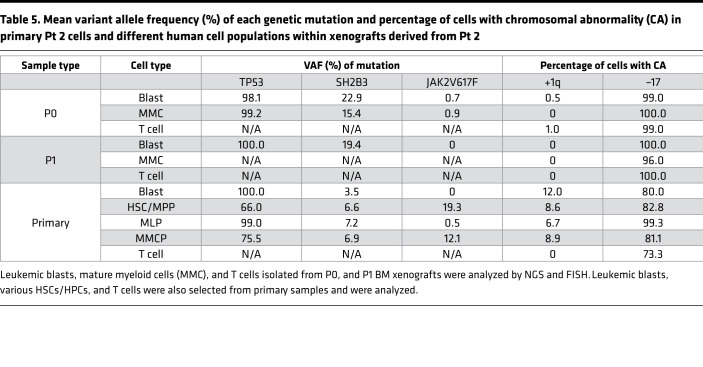
Mean variant allele frequency (%) of each genetic mutation and percentage of cells with chromosomal abnormality (CA) in primary Pt 2 cells and different human cell populations within xenografts derived from Pt 2

**Table 4:**
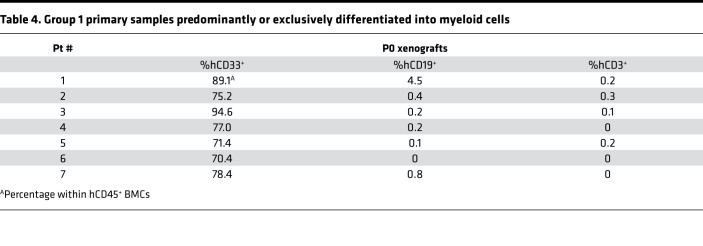
Group 1 primary samples predominantly or exclusively differentiated into myeloid cells

**Table 3:**
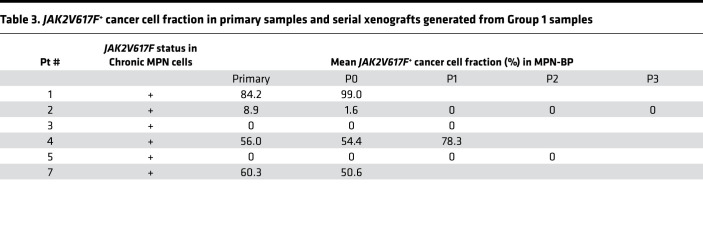
*JAK2V617F*^+^ cancer cell fraction in primary samples and serial xenografts generated from Group 1 samples

**Table 2:**
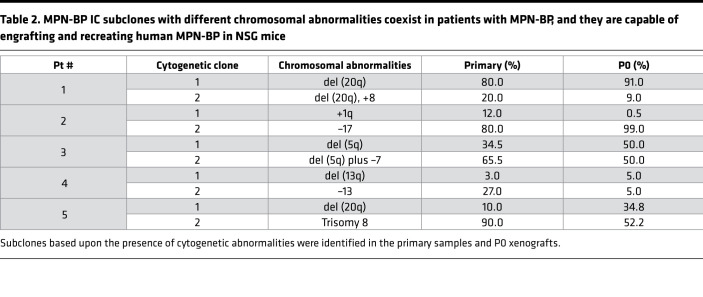
MPN-BP IC subclones with different chromosomal abnormalities coexist in patients with MPN-BP, and they are capable of engrafting and recreating human MPN-BP in NSG mice

**Table 1:**
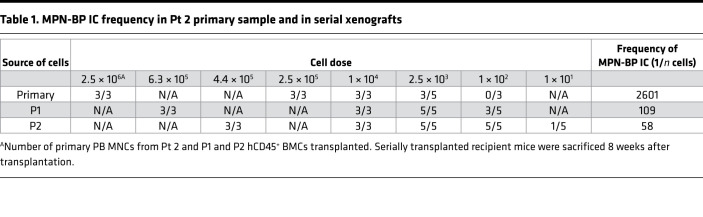
MPN-BP IC frequency in Pt 2 primary sample and in serial xenografts

## Version 1 03/08/2022

In-Press Preview

## Version 2 04/22/2022

Electronic publication
