# Supplementary material for: Characterization of disease-propagating stem cells responsible for myeloproliferative neoplasm–blast phase
Source: JCI Insight. 2022 Apr 22;7(8):e156534. doi: 10.1172/jci.insight.156534 (PMC9089790; doi:10.1172/jci.insight.156534)
Supplement: Supplemental data [file jciinsight-7-156534-s066.pdf]

Supplemental Material

Manuscript

Characterization of Disease-Propagating Stem Cells Responsible for Myeloproliferative Neoplasm-Blast Phase

Wang et al.

Supplemental methods

Capture based next generation sequencing (NGS) of a primary patient sample and samples from transplanted NSG mice (Pt 2)

With the leukemic blasts and hematopoietic stem cells and progenitor cells isolated from Pt 2 PB mononuclear cells and human cells present within xenografts following their transplantation into NSG mice, next generation sequencing (NGS) was performed using a panel which was comprised of 40 key DNA genes and a broad fusion panel of 29 driver genes that covers the most relevant targets in major myeloid malignancies (Oncomine™ Myeloid Research Assay, Thermo Fisher Scientific). Genomic DNA was isolated from each cell population using the AllPrep DNA/RNA Mini Kit (Qiagen) and quantified using a Synergy HTX Plate Reader spectrophotometer (BioTek, Model S1LFA) with Qubit dsDNA high-sensitivity reagents (Thermo Fisher Scientific). Sequencing libraries were prepared using 20ng of DNA as template and amplified according to the protocol established by Thermo Fisher Scientific for the Oncomine™ Myeloid Research Assay. Libraries were quantified using the KAPA Library Quantification kit for Ion Torrent (Roche)) and a LightCycler 480 instrument (Roche). Normalized libraries were pooled and templated using an Ion Torrent Chef and loaded onto an Ion Torrent 530 Sequencing Chip (Thermo Fisher Scientific). Sequencing was performed using the 400-cycle kit (Thermo Fisher Scientific) on an Ion Torrent S5 instrument (Thermo Fisher Scientific). Sequencing data was

aligned to hg19 and variants were called using Torrent Suite software **(**Thermo Fisher Scientific**)**. Variant call files were annotated using Ion Reporter **(**Thermo Fisher Scientific**)** and SnpEff (1) tools**.** Molecular variants were visually confirmed in aligned sequences using the Integrative Genomics Viewer (IGV) (2).

Supplemental reference

1. Li H. Exploring single-sample SNP and INDEL calling with whole-genome de novo assembly. *Bioinformatics.* 2012; 28: 1838-1844.
2. Robinson JT, et al. Variant Review with the Integrative Genomics Viewer (IGV). *Cancer Research.* 2017; 77: 31-34

Supplemental figures and figure legends

Supplemental Figure 1

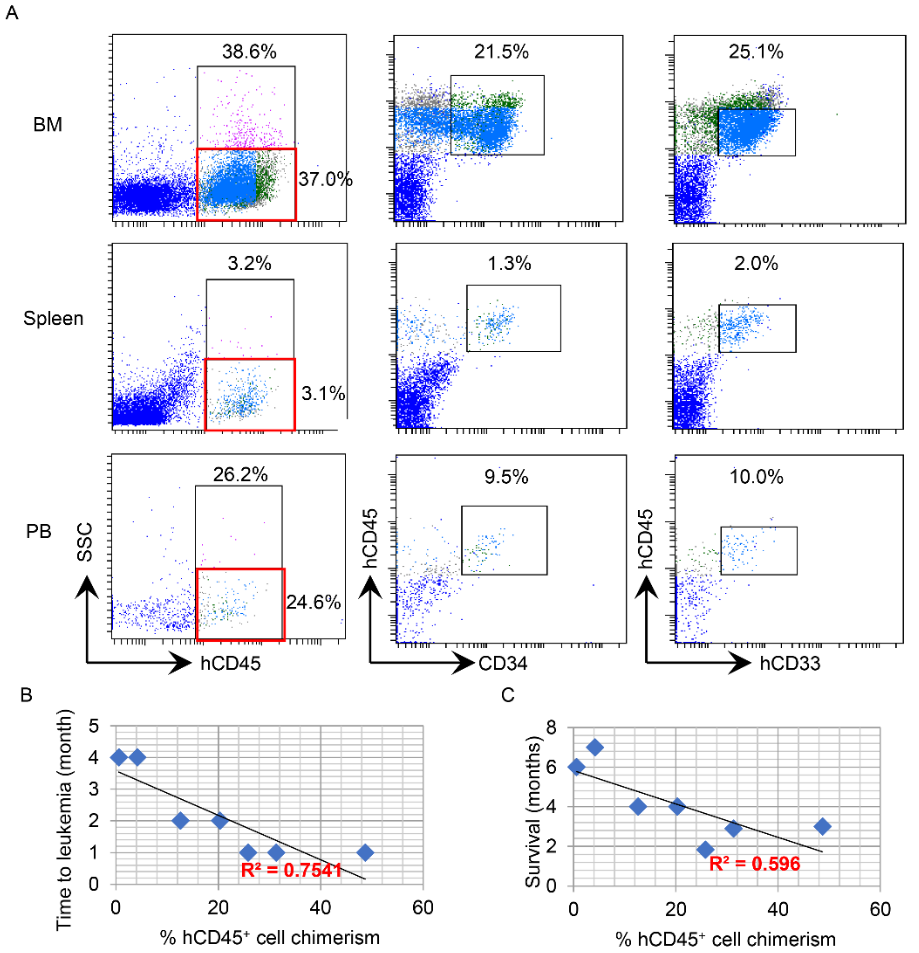

Supplemental Figure 1. A positive correlation between the efficiency of engraftment of MPN-AP/BP initiating cells (IC) and clinical outcomes of NSG mice engrafted with MPN AP/BP patient samples. (A) Flow cytometric analyses of BM, spleens and PB of NSG mice receiving PB $CD3^+$ cell-depleted mononuclear cells (MNC) from patients with MPN-AP/BP. Human cell engraftment {Left panel: human (h) $CD45^+$ cell chimerism is shown within black frame} and leukemic cell burden {percent of $hCD34^+$ cells (middle panel) and percent of $hCD45^{dim}CD33^+$ cells (right panel)} among viable nucleated cells in the BM, spleen and PB of a NSG mouse receiving a representative Pt 5 primary sample are shown. Upper numbers in left panel indicate the percent of $hCD45^+$ cells among viable nucleated cells. $SSC^{low}CD45^+$ blast population is shown within red frame in left panel which is further defined by expression pattern of hCD45 and hCD34 (middle panel) or hCD45 and hCD33 (right panel). PB: peripheral blood. **(B-C)** The association between MPN-BP cell chimerism and time to leukemia (B) and recipient mouse survival (C) of recipient mouse. X-axis indicates the % of $hCD45^+$ cell chimerism in the BM of mice 8-9 weeks after the transplantation with primary samples from Pts 1-4, 6-7, and 7 weeks after the transplantation with primary samples from Pt 5. Time to MPN-BP was determined by the time point at which ≥0.5% $hCD45^{dim}CD33^+$ or $hCD34^+$ leukemic blasts were detected in the PB of recipient mice. R^2: coefficient of determination. The degree of MPN-BP cell chimerism was inversely related to the time to leukemia initiation (B) and survival (C) in NSG recipient mice. 7 samples from Group 1 were analyzed.

Supplemental Figure 2

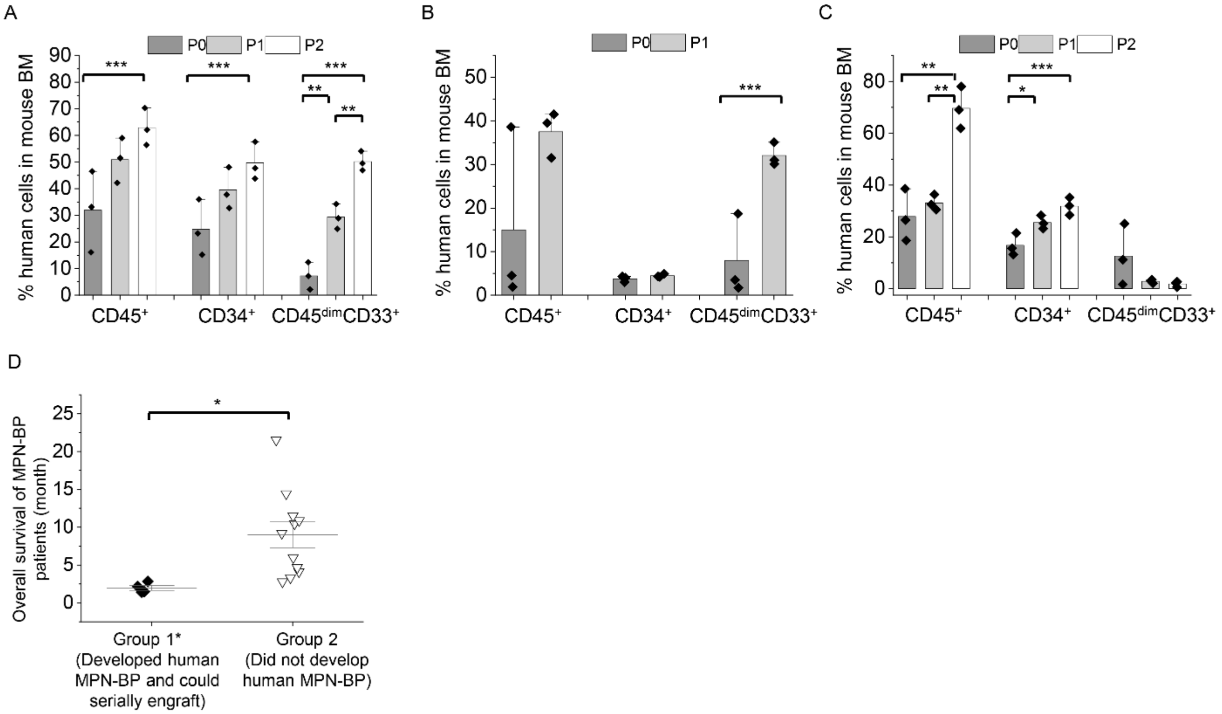

Supplemental Figure 2. The engraftment of MPN-BP ICs and their behavior following serial transplantation. (A-C) Serial xenotransplantation studies of primary samples from Pt 3 (A), Pt 4 (B) and Pt 5 (C). Human cell chimerism and leukemia cell burden (% of $hCD34^+$ and % of $hCD45^{dim}CD33^+$ cell) in serial recipient BM are shown. The mice were sacrificed 8-9 (A, B) or 7 (C) weeks after the transplantation. Bars indicate the mean in A-C. All data are represented as the mean ± SEM. Results of 3 mice receiving primary or xenograft samples are shown. *P<0.05, **P<0.01. ***P<0.001 by ANOVA. No. of $hCD34^+$ cells contained in transplanted grafts: A: Primary: 8.2×10^5; P0: 3.6×10^5; P1: 2.4×10^5. B: Primary: 3×10^5; P0: 2×10^5. C: Primary: 1.7×10^5; P0: 8.0×10^4; P1: 7.0×10^4. **(D)** Overall survival of patients with primary cells that were serially transplanted in NSG mice was significantly shorter than patients with primary cells that were not capable of recapitulating human MPN-BP in NSG mice. Group 1* include 4 patients (Pts 2-5) belonging to Group 1 with samples capable of serially engrafting and causing MPN-BP within 4 months in NSG mice. Group 2: n=11 different patient samples. * P=0.03 by ANOVA.

Supplemental Figure 3

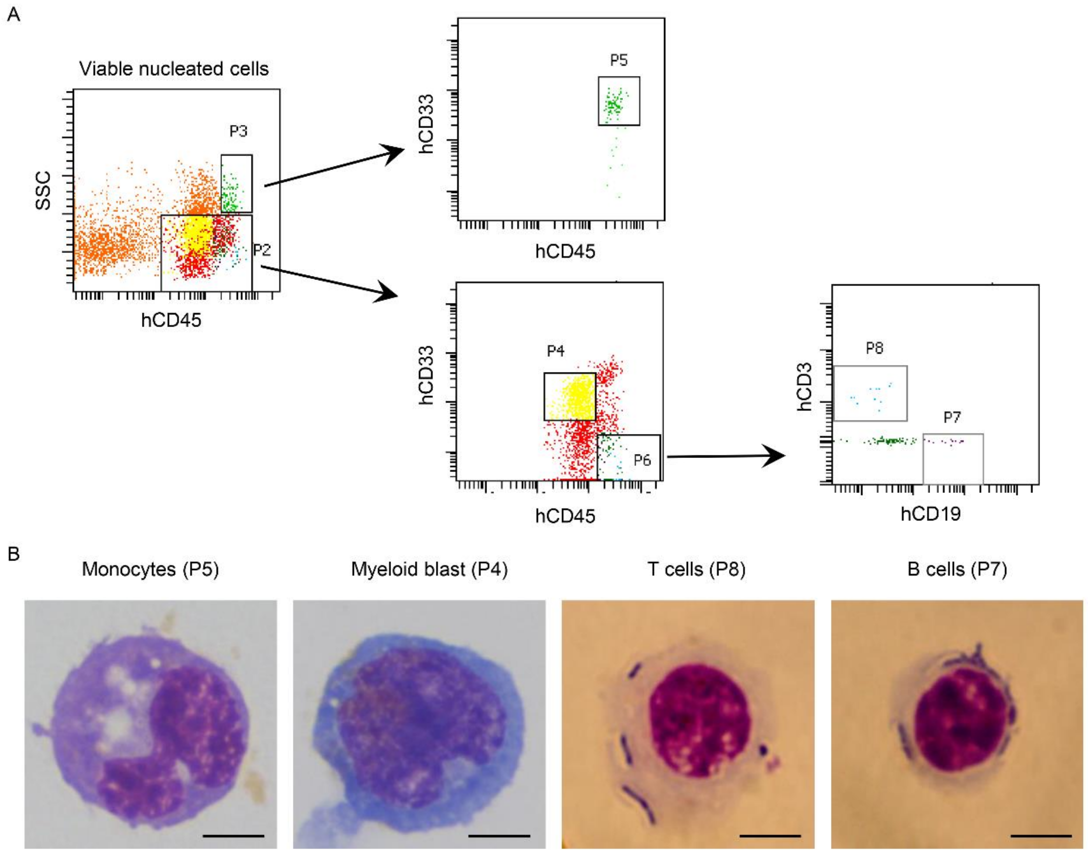

Supplemental Figure 3. Isolation of different cell populations from xenografts by FACS using a combination of human CD45, CD33, CD19 and CD3 mAbs. (A) FACS plots showing isolation of human monocytes ($SSC^{int/high}$ $CD45^{int/bright}$ $CD33^{+}$, P5), myeloid blasts ($SSC^{low}CD45^{dim}CD33^{+}$, P4), T cells ($SSC^{low}CD45^{int/bright}CD33^{-}CD3^{+}$, P8) and/or B cells ($SSC^{low}CD45^{int/bright}CD33^{-}CD19^{+}$, P7) from the BM cells (BMC) of a primary recipient mouse transplanted with a Group 1 patient primary sample (Pt 1). **(B)** Wright-Giemsa staining of human cells selected from the BMCs of a primary recipient mouse transplanted with a Group 1 patient primary sample (Pt 1). Flow cytometric analyses confirmed that the purity of myeloid blasts was 90% or greater. The purity of mature myeloid cells, and T/B cells as well as leukemic blasts was at least 90% by morphologic evaluation. Scale bars represent 5µm.

Supplemental Figure 4

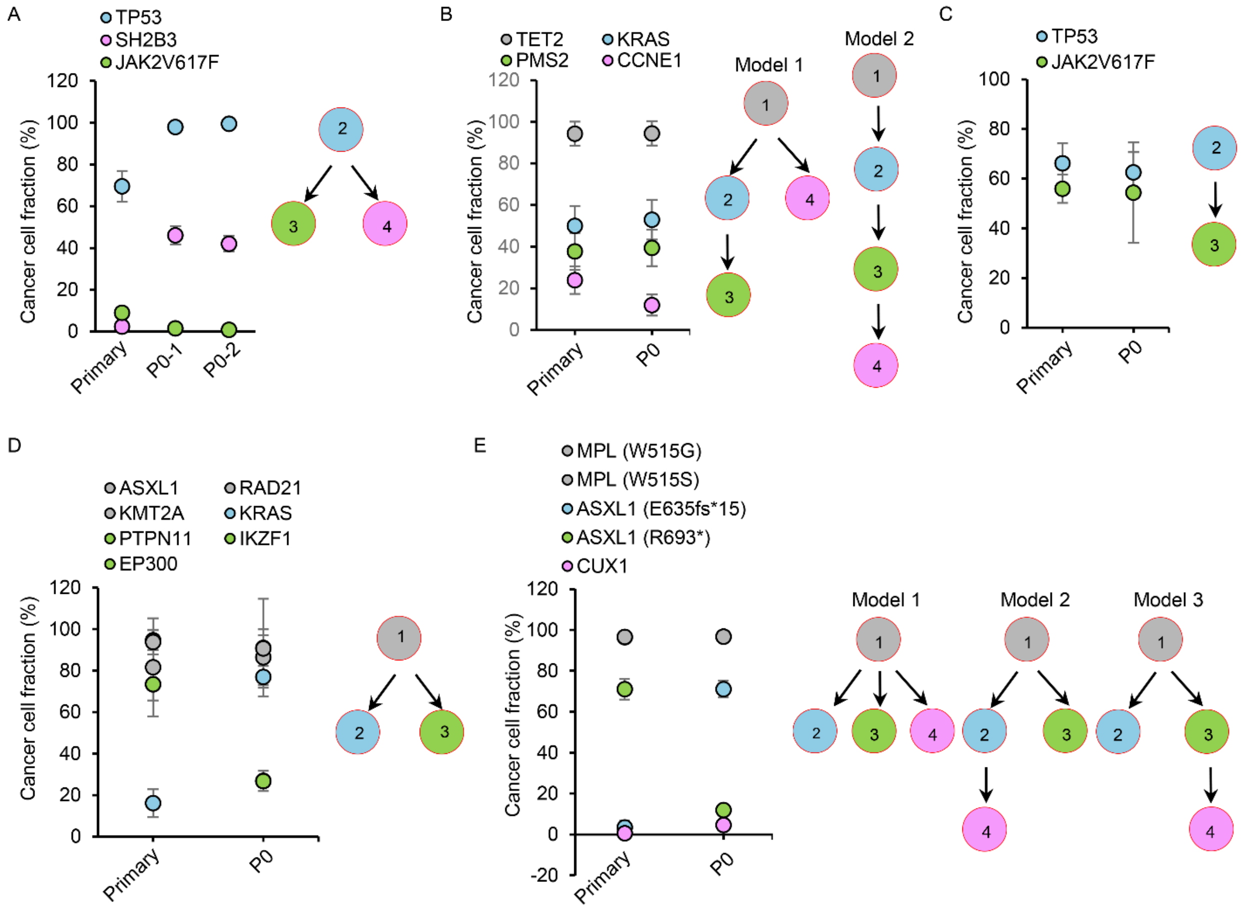

Supplemental Figure 4. In patients with MPN-AP/BP, multiple subpopulations of MPN-BP IC as defined by their mutational burden co-exist and are capable of engrafting and re-creating human MPN-BP in NSG mice. (A-E) The percentage of leukemic cells carrying each genetic mutation (cancer cell fraction, CCF) in primary samples and in the corresponding individual P0 xenografts (left scatter plot) and the inferred MPN-BP IC clonal hierarchy (right diagram) for Pt 2 (A), Pt 3 (B), Pt 4 (C), Pt 5 (D), and Pt 6 (E) belonging to Group 1. Mutations clustered together are indicated by the same color. Each circle represents a clone. Throughout each panel, the founder clone containing mutations indicated in dark grey in the left scatter plot is also shown in dark grey and is indicated in #1. The founder clone was not identified in Pts 2, 4 (A, C). Each subclone which is evolved from the parent clone is indicated by the circle of the same color as that denotes the mutations newly acquired in the left scatter plot and is indicated by the #1-4. In Pts 2, 4, and 5, Clonevol identified only one consensus model, while in Pts 3 and 6, this same program inferred several consensus models to explain the clonal hierarchy of MPN-BP ICs.

Supplemental Figure 5

A

Pt 2	Clone 1 +1q	Clone 2 -17
Primary	12.0	80.0
P0	0.5	99.0
P1	0.0	100.0
P2	0.0	96.0
P3	0.0	100.0

B

Pt 3	Clone 1 del (5q)	Clone 2 del (5q) plus -7
Primary	34.5	65.5
P0	50	50
P1	20	80

C

Pt 4	Clone 1 del (13q)	Clone 2 -13
Primary	3	27
P0	5	5
P1	15.4	0

D

Pt 5	Clone 1 del (20q)	Clone 2 Trisomy 8
Primary	10	90
P0	34.8	52.2
P1	0	100
P2	0	100

Supplemental Figure 5. MPN-BP ICs with different chromosomal abnormalities undergo clonal competition following serial transplantation. (A-D) The percentage of leukemia cells with different chromosomal abnormalities in primary patient cells and serial xenografts is shown. For Pt 4, primary mononuclear cells were analyzed by FISH. For other primary samples and all xenograft samples, 10-100 sorted leukemic blast cells/sample were analyzed. MPN-BP ICs containing -17 or -7 possessed a competitive growth advantage.

Supplemental Figure 6

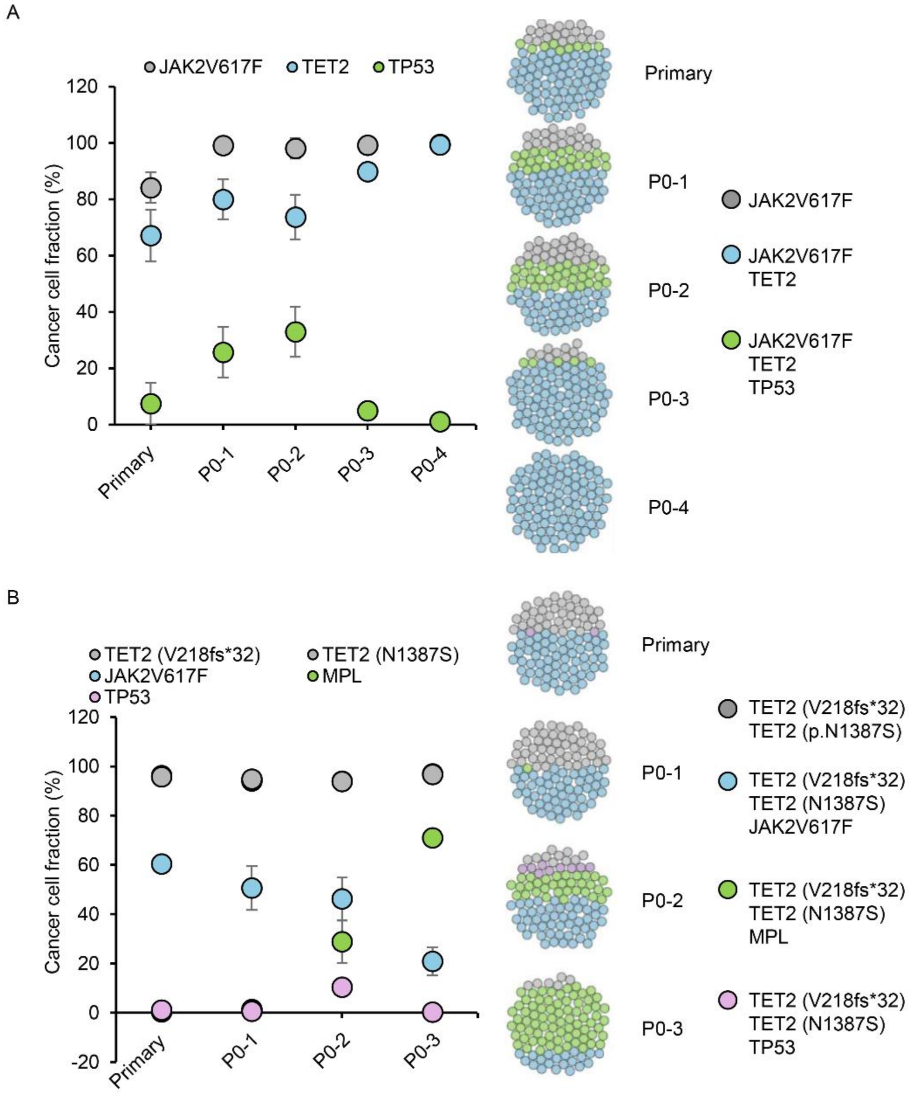

Supplemental Figure 6. Acquisition of *JAK2V617F* alone does not confer a proliferative advantage to the MPN-BP ICs. (A-B) The percentage of leukemia cells containing each genetic mutation (Cancer cell fraction, CCF, left scatter plot) and clonal and subclonal populations (right sphere of cells) in Pt 1 (A) and Pt 7 (B) primary sample and multiple individual P0 xenografts derived from these two patients' primary samples are shown. In each of 4 P0 xenografts derived from Pt 1, *JAK2V617F* CCF of the founder clone was either unchanged (P0-1 and P0-2) or decreased (P0-3 and P0-4) as compared to that present in the primary sample (A). For Pt 7, *JAK2V617F* CCF was also reduced in each of P0 xenografts. These observations suggest that acquisition of *JAK2V617F* alone does not confer proliferative advantage to the MPN-BP IC clone/subclone in these two patients.

Supplemental Figure 7

A

Pt 1

		JAK2V617F	TET2	TP53	del (20q)	del (20q) plus +8
P0-3	Blast	99.5	88.1	2.4	91.0	9.0
	MMC	99.8	75.2	3.7	79.0	21.0
	B Cell	100.0	85.4	8.4	84.0	16.0
	T Cell	99.7	92.9	8.3	88.0	12.0
P0-4	Blast	99.8	99.5	0.5	97.0	3.0
	MMC	99.8	97.2	0.7	100.0	0.0
	B Cell	97.0	99.7	8.4	N/A	N/A
	T Cell	94.4	91.7	6.2	80.0	15.0

B

Pt 3

		TET2	PMS2	KRAS	del (5q)	del (5q) plus -7
P0	Blast	49.9	30.3	44.4	50.0	50.0
	MMC	50.6	26.9	49.1	42.9	57.1
P1	Blast	50.5	28.4	90.2	20.0	80.0
	MMC	46.0	29.1	91.9	33.3	66.7

C

Pt 4

		TP53	JAK2V617F	del (13q)	-13
P0	Blast	55.2	36.6	5.0	5.0
	MMC	9.0	26.5	4.5	22.7
	B Cell	0.3	3.8	1.0	4.0
P1	Blast	99.5	39.2	50.0	0.0
	MMC	98.6	35.5	50.0	0.0

Supplemental Figure 7. MPN-BP ICs retain the capacity to differentiate into mature myeloid and lymphoid cells. (A-C) Mean variant allele frequency (%) of each genetic mutation and percent of cells with chromosomal abnormalities in different cell populations within xenografts derived from Pt 1 (A), Pt 3 (B), Pt 4 (C) are shown. Leukemic blasts, mature myeloid cells (MMC), T cells and/or B cells isolated from P0 and P1 BM xenografts were analyzed by NGS and FISH.

Supplemental Figure 8

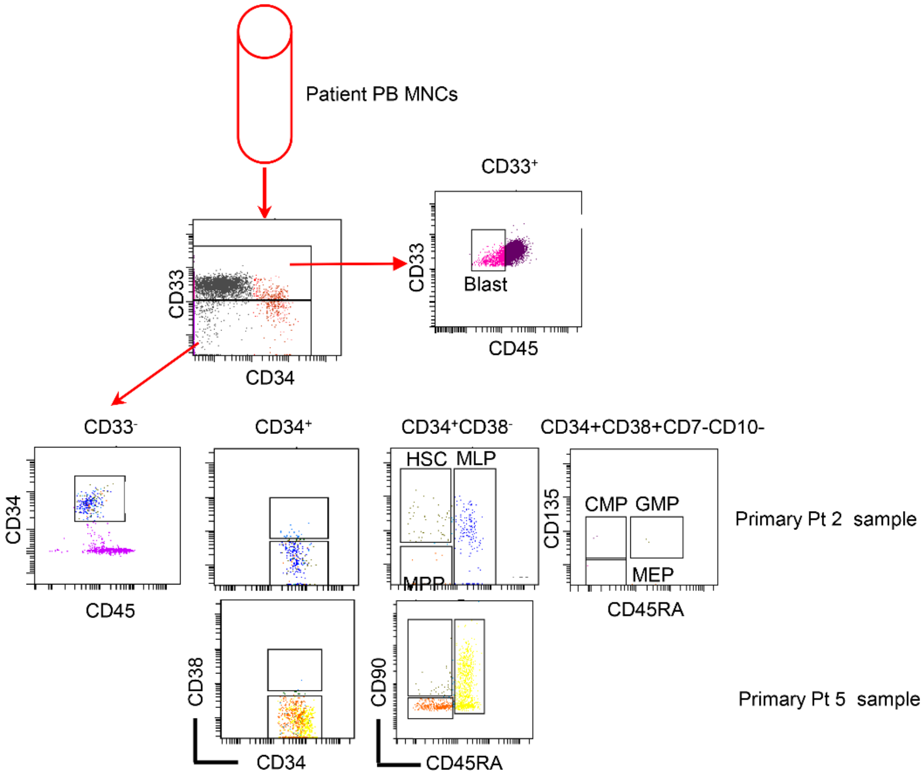

Supplemental Figure 8. Sorting strategy used to isolate hematopoietic stem cell (HSC) and various progenitor cell populations and leukemic blasts from primary samples from Pts 2 and 5. Primary samples were sorted into $CD45^{dim}CD33^{+}$ blasts as well as $CD33^{-}$ HSCs and various progenitor cell subpopulations. These cell subpopulations were then transplanted into NSG mice. Capture based targeted deep sequencing and FISH were performed when sufficient number of cells in the various cell subpopulations were available. PB: peripheral blood; MPP: multipotent progenitor; MLP: multilymphoid progenitor; CMP: common myeloid progenitor; GMP: granulocyte-monocyte progenitor; MEP: megakaryocyte-erythroid progenitor. Since very limited numbers of $CD34^{+}CD38^{+}$ cells were present in the sample from Pt 5, we were unable to isolate CMPs, GMPs or MEPs.

Supplemental Figure 9

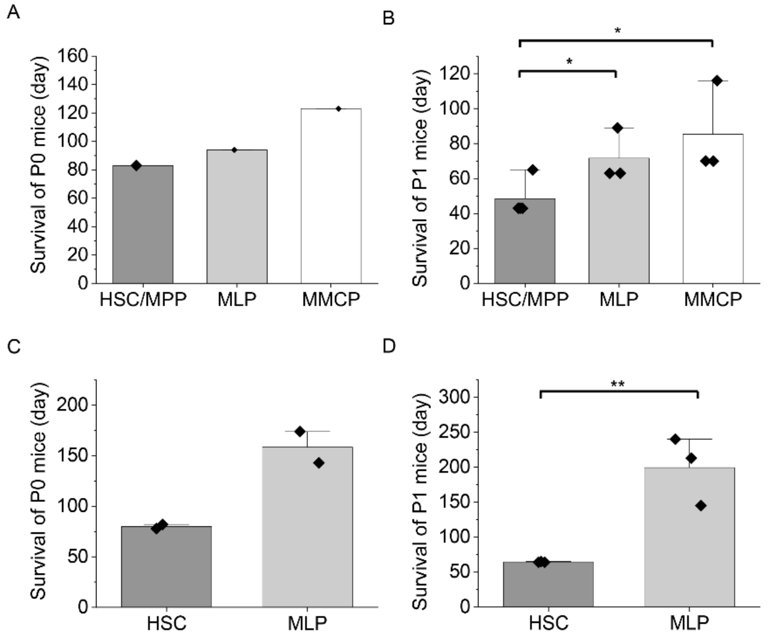

Supplemental Figure 9. MPN-BP IC with the phenotype of a HSC/MPP leads to the development of a more fulminant form of MPN-BP in NSG mice. (A, C) Survival of the P0 recipients receiving FACS-sorted HSCs/MPPs, MLPs and MMCPs from Pt 2 (A) or HSCs, MLPs from Pt 5 (C). (A): n each =1 mouse. (C): n each=2 mice. (B, D) Survival of the secondary (P1) recipients receiving BM cells collected from P0 recipients that received purified HSCs/HPCs from Pt 2 (B) or Pt 5 (D). (B): HSC/MPP: n=4 mice. MLP, MMCP: n each =3 mice. (D): n each =3 mice. Bars indicate the mean in A-D. Data are represented as the mean ± SEM. Statistical significance was determined using ANOVA. *P<0.05, **P<0.01.

Supplemental Table 1. Clinical characteristics of patients with MPN-AP/BP

Pt #	Prior MPN	Driver mutations in chronic MPN	Current Diagnosis	Karyotype in MPN-AP/BP Clone 1	Karyotype in MPN-AP/BP Clone 2	Mutations in MPN-AP/BP
1	PV-MF	*JAK2V617F+*	BP	46, XX, der(1)t(1;7)(p22;q21.1), del(7)(q21.2), der (7)del(7)(q21.2)t(1;7)(p22;q21.1),del(20)(q11.2q13.1) [12]*	47, XX, der(1)t(1;7)(p22;q21.1), del(7)(q21.2),der(7)del(7)(q21.2)t(1;7)(p22;q21.1),+8,del(20)(q11.2q13.1) [3]	*JAK2V617F*, *TET2* (I1873T), *TP53* (I251F)
2	PV-MF	*JAK2V617F+*	BP	46, XX, +1, der(1;18)(q10;q10) [15]	44, XX, -12, -17, add(20)(q12) [2]	*JAK2V617F*, *TP53* (E343fs*2), *SH2B3* (L377P)
3	PV	*JAK2V617F+*	BP	45, XX, -3, del(5)(q13q33),-7, add(9)(p24),-14,add(18)(p11.2) [18	46, XX, -3, del(5)(q13q33),-7,add(9)(p24),add(18)(p11.2) [2]	*KRAS* (p.G13D), *TET2* (E711*), *PMS2* (L731fs*3), *CCNE1* (E4*)
4	ET	*JAK2V617F+*	BP	45, XY, add(1)(p13),-13, -15, -19, add(21)(q22) [17]	46, XY [3]	*JAK2V617F*, *TP53* (P128fs*42)
5	AP-MPN	*JAK2V617F+*	BP	47, XY, +8 [18]	46, XY, del(20)(q11.2q13.1) [2]	*KRAS* (G12C), *KMT2A* (S2231fs*7), *PTPN11* (A72T), *ASXL1* (T848fs*19), *IKZF1* (N159S), *RAD21* (M489I), *EP300* (R613P)
6	ET-MF	*MPL*	AP	46, XY		*MPL* (W515G), *MPL* (W515S), *ASXL1* (E635fs*15), *ASXL1* (R693*), *CUX1* (Q1032*)
7	PV	*JAK2V617F+*	BP	46, XY		*JAK2V617F*, *MPL* (Y591N), *TET2* (V218fs*32), *TET2* (N1387S), *TP53* (I195T)
8	ET-MF	*JAK2V617F+*	AP	46, XY		*JAK2V617F*, *EZH2* (D664V)
9	PMF	Triple Negative	AP	46, XY		*MPL* (R592*), *MPL* (V437_L438insWSSNKCPQTSDNTGEGHQDWKV), *SF3B1* (K666N), *BCOR* (A1657fs*2)
10	AP-MPN	*JAK2V617F+*	AP	46, XY		*JAK2V617F*, *TP53* (R248W)
11	ET	Triple Negative	BP	46, XY		*CCND2* (P281R), *DNMT3A* (R882C), *SETBP1* (G870S), *U2AF1* (S34Y), *SH2B3* (H137fs*60), *CUX1*(F623delF)
12	PV	*JAK2V617F+*	BP	46, XY		*JAK2V617F*, *TET2* (H1386fs*15), *TET2* (F519fs*14), *RUNX1* (G165V), *RUNX1* (H105Y), *SRSF2* (P95H), *IDH1* (R132C)
13	PV	*JAK2V617F+*	AP	46, XY[18]	43, XY, -5, -7, add(16)(p12), -17, -19,+mar[2]	*JAK2V617F*, *TP53* (R280G), *ASXL1* (R965*)
14	PMF	N/A	AP	46, XY		*JAK2V617F*, *ASXL1*(Q748*), *SRSF2* (P95A), *STAG2(R614*)*, *SF3B1*(K666N), *CEBPA* (D301fs*18)
15	PMF	N/A	AP	45, XX, del(5)(q31q35), del(14)(q11.2q13),-17, der(19) t(17;19)(q11.2;p13.1)t(5;17) (q31;q25) [9]	46, XX, t(4;14;19)(q21;q11.2;q13) [3]	*MPL* (H499fs*45), *ARID1A* (Y1096C)
16	PV	*JAK2V617F+*	BP	46, XY		*JAK2V617F*, *RUNX1* (Q335fs*255), *PDS5B* (unknown)
17	PV	*JAK2V617F+*	BP	46, XY		*JAK2V617F*, *TP53* (L257R),
18	PMF	N/A	BP	Other#		*MPL* (H624D), *CALR* (K374fs*48), *U2AF1* (Q157P), *RUNX1* (F163V)

*The number in brackets denotes the number of cells having the indicated karyotype among the 20 cells evaluated. #Interphase FISH results for recurrent chromosomal abnormalities -5/del(5q), -7/del(7q) and +8 were normal. N/A: Not available. PV: polycythemia vera, ET: Essential thrombocythemia, PMF: Primary myelofibrosis. Symbols for genes are italicized. See also Supplemental Table 11 for the variant allele frequency and other information of each mutation.

Supplemental Table 2. Engraftment of MPN-AP/BP cells in NSG mice

	**Group 1 (Develop human MPN-BP in NSG mice)**	**Group 2 (Do not develop human MPN-BP in NSG mice)**	
		Engraft in NSG mice	Do not engraft in NSG mice
Patient#	1-7	8-12	13-18
MPN-BP	6	2	3
MPN-AP	1	3	3
% Human chimerism in NSG mice (>0.1% $hCD45^+$ cells in BM)	$35.8±7.3^*$	$1.9±0.9^*$	0
Time to MPN-BP in NSG mice (Month)	1-4	-	-
Overall survival of NSG mice (Month)	$4.1±0.7^*$	>7	>7

NSG mice transplanted with $CD3^+$ cell-depleted MNCs from individual patients were regarded as having developed human MPN-BP if at least 20% $hCD45^{dim}CD33^+$ or $hCD34^+$ cells or at least 20% blasts as detected by flow cytometry and/or morphological examination were present in the marrow and spleen of recipient mice. Mice receiving Group 2 samples were sacrificed at 4-7 months after the transplantation. *Data are represented as mean ± SEM.

Supplemental Table 3. Frequency of MPN-BP ICs in primary MPN AP/BP MNCs

Group 1 Pt #	Group 1 MPN-BP IC frequency (1/*n* cells)	Group 2 Pt #	Group 2 MPN-BP IC frequency (1/*n* cells)
1	2684	8	41704
2	2600	9	7266979
3	2700	12	1397371
4	25749		
5	6438		
6	58011		
7	92043		
Mean	27175	Mean	2902018

Peripheral blood $CD3^+$ cell-depleted mononuclear cells (MNCs) from Group 1 (7) and Group 2 (3) patients with MPN-AP/BP were transplanted into NSG mice using a limiting diluting analysis (1×10^2-1×10^7, 3-5 mice/group). The frequency of MPN-BP ICs among $CD3^+$ cell-depleted MNCs from each sample was calculated using Poisson statistics with L-Calc software. P=0.06, MPN-BP IC frequency of Group 1 vs. MPN-BP IC frequency of Group 2.

Supplemental Table 4. MPN-BP IC frequency in Pt 5 primary sample and in serial xenografts

**Source of cells**	**Cell dose**								**Frequency of MPN-BP IC (1/n cells)**
	**$2.5\times10^{6*}$**	**6.3×10^{5}**	**4.4×10^{5}**	**2.5×10^{5}**	**1×10^{4}**	**2.5×10^{3}**	**1×10^{2}**	**1×10^{1}**	
Primary	3/3	N/A	N/A	3/3	3/3	3/5	0/3	N/A	2601
P1	N/A	3/3	N/A	N/A	3/3	5/5	3/5	N/A	109
P2	N/A	N/A	3/3	N/A	3/3	5/5	5/5	1/5	58

*: Number of primary PB MNCs from Pt 2 and $hCD45^{+}$ BMCs from P1 and P2 recipients transplanted. Serially transplanted recipient mice were sacrificed 8 weeks after transplantation and an analysis of human cell engraftment and the leukemic cell burden in the BM, spleen and PB was performed. N/A: not available.

Supplemental Table 6. Mean variant allele frequency (%) of each genetic mutation and percent of cells with chromosomal abnormality (CA) in primary Pt 5 cells and different human cell populations within xenografts derived from Pt 5

Sample type	Cell type	VAF (%) of mutation							% of cells with CA	
		ASXL1	*RAD21*	*KMT2A*	*KRAS*	*PTPN11*	*IKZF1*	*EP300*	del (20q)	Trisomy 8
P0	Blast	48.8	43.3	45.2	77.4	12.2	13.9	12.5	34.8	52.2
	MMC	48.9	43.8	42.7	96.4	2.6	0.8	1.2	52.0	36.0
	T Cell	65.0	48.3	65.4	100.0	0	0.9	0	66.7	0
P1	Blast	31.0	37.6	35.4	100.0	0	0	0	0	100.0
	MMC	47.3	42.7	43.9	100.0	0.5	0	0.2	0	100.0
Primary	Blast	64.9	22.3	44.5	9.6	40.6	36.4	33.3	10.0	90.0
	HSC	48.1	30.1	46.0	19.0	42.0	43.7	35.2	10.3	85.3
	MLP	46.7	28.9	43.4	1.5	41.0	51.8	42.7	0	94.6

BMCs of NSG mice 7 weeks after transplantation of Pt 5 PB $CD3^+$ cell-depleted MNCs were analyzed by flow cytometry. Leukemic blasts, mature myeloid cells (MMC) and T cells isolated from P0, and P1 BM xenografts were analyzed by NGS and FISH. Leukemic blasts, HSCs and MLPs were also selected from primary samples and were analyzed. HSC: hematopoietic stem cell; MLP: multilymphoid progenitor.

Supplemental Table 7. Differential pattern of purified primary HSC/HPC subpopulations from Pt 5 in P0 NSG recipients

Cells transplanted	Xenografts		
	%hCD33$^+$	%hCD19$^+$	%hCD3$^+$
HSC	72.5*	0.2	0.1
MLP	77.8	0.1	0

*: % within hCD45$^+$ BMCs

Supplemental Table 8. Mean variant allele frequency (%) of each genetic mutation and percent of cells with chromosomal abnormality (CA) in different human cell populations within P0 xenografts derived from purified primary HSCs and MLPs from Pt 5

**Cell injected**	**Human cell in P0 xenograft**	**VAF (%) of mutation**							**% of cells with CA**	
		ASXL1	*RAD21*	*KMT2A*	*KRAS*	*PTPN11*	*IKZF1*	*EP300*	del (20q)	Trisomy 8
HSC	Blast	51.1	28.5	47.8	4.5	51.6	50.8	39.3	25.8	71.0
	T cell	N/A	N/A	N/A	N/A	N/A	N/A	N/A	16.0	76.0
MLP	Blast	48.1	34.5	46.9	0	46.5	49.3	49.4	0	83.3
	B cell	N/A	N/A	N/A	N/A	N/A	N/A	N/A	0	84.6

HSC: hematopoietic stem cell; MLP: multilymphoid progenitor; N/A: not available.

Supplemental Table 9. Mutational profile of primary cells from Pt 8 and hCD45$^+$ cells in P0 NSG recipient

	VAF (%)	
	EZH2 (D664V)	*JAK2V617F*
Primary cells	46.1	38.3
hCD45$^+$ cells of P0 NSG recipient	47.0	35.8

Primary mononuclear cells of Pt 8 (Group 2) and hCD45$^+$ cells isolated from the BM of P0 recipient mice were analyzed by NGS and FISH. VAF: Variant allele frequency.

Supplemental Table 10. Reagents utilized in flow cytometric analyses, immunohistochemical analyses and fluorescence in situ hybridization

Reagents	Source	Identifier
APC Mouse Anti-Human CD45	BD Pharmingen	Cat# 555485
APC Mouse Anti-Human CD34	BD Pharmingen	Cat# 555824
PE Mouse Anti-Human CD33	BD Pharmingen	Cat# 555450
Alexa Fluor®488 Mouse Anti-Human CD14	BD Pharmingen	Cat# 557700
FITC Mouse Anti-Human CD15	BD Pharmingen	Cat# 562370
FITC Mouse Anti-Human CD41a	BD Pharmingen	Cat# 340929
FITC Mouse Anti-Human CD19	BD Pharmingen	Cat# 555412
FITC Mouse Anti-Human CD3	BD Pharmingen	Cat# 555339
PerCP-Cy™5.5 Mouse Anti-Human CD33	BD Pharmingen	Cat# 341650
FITC Mouse Anti-Human CD45	BD Pharmingen	Cat# 555482
Per-Cy™7 Mouse anti-Human CD45RA	BD Pharmingen	Cat# 560675
PE Mouse Anti-Human CD90	BD Pharmingen	Cat# 555596
BV510 Mouse Anti-Human CD7	BD Pharmingen	Cat# 563650
Per-Cy™5 Mouse Anti-Human CD10	BD Pharmingen	Cat# 555376
PE Mouse Anti-Human CD135	BD Pharmingen	Cat# 558996
APC Mouse Anti-Human CD3	BD Pharmingen	Cat # 555342
APC/Cyanine7 Anti-Human CD38	BioLegend	Cat# 303534
Anti-CD34, Clone QBend/10	Biogenex	Cat # AM236-10M
Rabbit Anti-Human CD3	DAKO	Cat# A045201-2
Recombinant Anti-Myeloperoxidase antibody	Abcam	Cat# ab134132
Recombinant Anti-Human CD19	Abcam	Cat# ab134114
Recombinant Anti-Human CD79a	Abcam	Cat# ab79414
CEP 8 (D8Z2)	Abbott Molecular	Cat# 06J37-018
LSI TP53	Abbott Molecular	Cat# 05J52-011
CEP 17 (D17Z1)	Abbott Molecular	Cat# 06J37-027
LSI D20S108	Abbott Molecular	Cat# 05J47-011
